# Quantitative scoring of differential drug sensitivity for individually optimized anticancer therapies

**DOI:** 10.1038/srep05193

**Published:** 2014-06-05

**Authors:** Bhagwan Yadav, Tea Pemovska, Agnieszka Szwajda, Evgeny Kulesskiy, Mika Kontro, Riikka Karjalainen, Muntasir Mamun Majumder, Disha Malani, Astrid Murumägi, Jonathan Knowles, Kimmo Porkka, Caroline Heckman, Olli Kallioniemi, Krister Wennerberg, Tero Aittokallio

**Affiliations:** 1 Institute for Molecular Medicine Finland (FIMM), University of Helsinki, Helsinki, Finland; 2 Hematology Research Unit, Helsinki University Central Hospital (HUCH), Helsinki, Finland

## Abstract

We developed a systematic algorithmic solution for quantitative drug sensitivity scoring (DSS), based on continuous modeling and integration of multiple dose-response relationships in high-throughput compound testing studies. Mathematical model estimation and continuous interpolation makes the scoring approach robust against sources of technical variability and widely applicable to various experimental settings, both in cancer cell line models and primary patient-derived cells. Here, we demonstrate its improved performance over other response parameters especially in a leukemia patient case study, where differential DSS between patient and control cells enabled identification of both cancer-selective drugs and drug-sensitive patient sub-groups, as well as dynamic monitoring of the response patterns and oncogenic driver signals during cancer progression and relapse in individual patient cells *ex vivo*. An open-source and easily extendable implementation of the DSS calculation is made freely available to support its tailored application to translating drug sensitivity testing results into clinically actionable treatment options.

Cell-based compound screening provides a rich functional readout for many biomedical applications. In cancer research, the possibility to profile cellular responses to an extensive collection of anti-cancer compounds enables a systematic means to repurpose existing drugs to new indications, identify druggable vulnerabilities in various types of cancer cells and to functionally investigate cellular pathways behind drug sensitivity or resistance. Recent studies have successfully explained or even predicted drug responses by means of genetic aberrations or other genomic biomarkers in wide panels of cancer cell lines^1^,^2^,^3^,^4^. Similar large-scale drug testing efforts in primary cancer samples are increasingly being carried out to enable functional investigation of cellular addictions in individual cancer patients; for instance, to predict pathway dependencies and to identify potential therapeutic options for leukemia patients^5^,^6^. Systematic profiling of the relative activity of hundreds or thousands of drugs at several concentrations in a large number of cancer samples or cell types facilitates the stratification of cancer patients and disease subtypes, as well as development of personalized treatment strategies for clinical applications.

However, high-throughput drug testing experiments often result in high-dimensional sample-dose-response matrices, with inherent measurement noise and technical variability, which hinders many downstream analyses, such as those aimed at detecting differential drug sensitivities or clustering of patients and/or drugs based on their selective response patterns. To provide quantitative information about the degree of drug efficacy in a given sample, the dose dimension of these matrices is often summarized into single response parameter estimated form dose-response models, such as IC_50_ or EC_50_ (half-maximal inhibitory/effective concentration)^1^,^2^,^3^,^5^,^7^,^8^. Although proven sufficient in many applications, any single model parameter can capture only limited information about the differences in the response patterns^9^, especially when comparing cancer and normal cells. Recently, an ‘Activity Area' metric was used to estimate both the efficacy and potency of 24 compounds in hundreds of cancer cell lines^3^. This type of discrete approximation, based on summing up the observed responses at each dose level, was shown to perform well under controlled *in vitro* settings with relatively densely-sampled concentration ranges and narrow bioactivity spectra^3^.

Here, we developed and implemented a quantitative scoring approach, named drug sensitivity score (DSS), which captures and integrates the multiparametric dose-response relationships into a single metric to identify selective drug response patterns between cancer and control cells, rather than scoring drug activity in cancer cells alone. Analytic integration of the area under the non-linear dose-response model combines the advantages of both the model-based and area-based response calculations. Applications of DSS to drug sensitivity testing of acute myeloid leukemia (AML) patient cells *ex vivo* demonstrated its improved performance, also when profiling larger compound panels and broader bioactivity spectra at sparsely-sampled dose levels (10,000-fold range) in fresh, primary cells. Several case studies in *in vitro* models from the Cancer Cell Line Encyclopedia (CCLE) resource^3^ also supported the applicability of the DSS metric to various experimental settings and application cases, where the aim is to identify both sensitive and selective drug response patterns. To promote its application to the future drug testing studies, we have made publicly available an open-source and easily extendable implementation of the model-based DSS calculations in the form of a stand-alone R-package.

## Results

**Figure 1:**
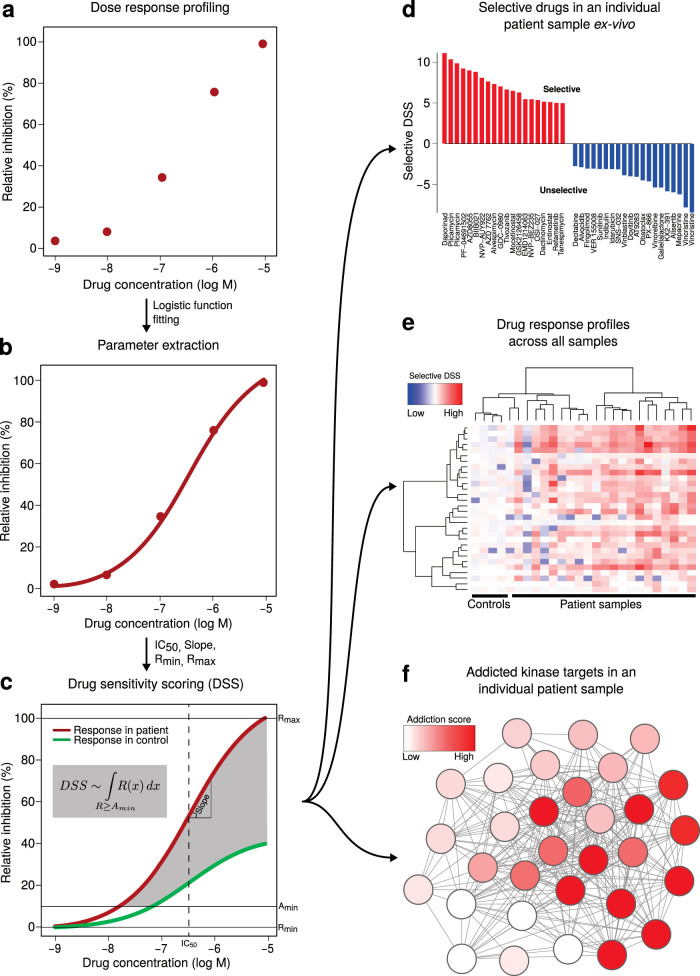
Implementation of the drug sensitivity scoring (DSS) pipeline in the AML samples. (a) Each compound was tested in a dose response series in 10-fold dilutions at 5 different concentrations (typically 1–10,000 nM). The response readout (CellTiter-Glo reagent) was normalized using positive and negative controls on each dose plate to provide the response measure (relative inhibition %). (b) Dose-response parameters estimated through logistic function model include IC_50_ (half-maximal inhibitory concentration), slope of the curve at IC_50_, and the bottom and top asymptotes of the curve (R_min_ and R_max_). (c) Schematic illustration of the differential DSS calculation (dDSS, the grey area). The two dose-response curves show clearly differential activity patterns, yet their relative IC_50_ is equal, showing an example in which IC_50_ is not informative enough for detecting selective responses in patient samples. *Inset*: analytic calculation of the DSS statistic as an integral over the dose range where the drug response exceeds a given minimum activity level A_min_. (d) Waterfall plots of the individual dDSS profiles enable identification of cancer-selective drugs for a given patient sample. (e) Heatmap plots of the dDSS profiles over all the samples enable identification of drug-sensitive patient sub-groups. dDSS for the control samples reflect the variability among the control sample responses. (f) Network maps of the kinases the particular sample is addicted to enable identification of oncogenic driver signals.

Our quantitative scoring approach is based on closed-form integration of the area under the estimated dose-response curve (AUC; Figure 1a); the generic modeling approach can be used in the context of standard logistic, sigmoidal or Hill slope response functions (Figure 1b). The continuous model estimation and interpolation effectively summarize the complex dose-response relationship into a single response metric, named DSS (Supplementary Fig. 1b). More formally, if *R*(*x*) models the normalized drug response at a concentration *x*, then the integral response *I* over the dose range that exceeds a given minimum activity level, A_min_, is calculated analytically as a continuous function of multiple parameters of the non-linear response model, including its slope at IC_50_ as well as the top and bottom asymptotes of the response (R_max_ and R_min_).: 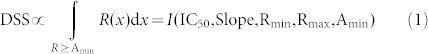
Importantly, differential DSS (dDSS) quantifies the selective response of cancer cells, relative to that of control cells, when control samples are available; dDSS is calculated by the difference between drug response quantified in patient cells (patient DSS) and the average drug response of control samples (controls DSS) (Figure 1c). To discriminate those compounds which are effective at higher concentrations only (potential toxic off-target responses), and to favor those that show potency over a relative wide therapeutic window, the analytic AUC calculation (referred to as DSS_1_) was further normalized by the logarithm of the top asymptote R_max_(DSS_2_) and by the dose range over which the response exceeds the activity threshold A_min_ (DSS_3_), respectively (mathematical derivation of the closed-form solutions when using four-parameter logistic response model is given in Supplementary Methods). The DSS R-package and its source code are freely available at https://dss-calculation.googlecode.com/svn/trunk/.

### DSS calculation improves drug response profiling in primary leukemic cells

We initially developed and implemented the DSS calculation in the context of our ongoing drug sensitivity and resistance testing (DSRT) program, with the aim to provide informed choices for clinicians on the treatment of relapsed or chemorefractory acute myeloid leukemia (AML) patients based on the *ex vivo* DSRT results of the patient cells^6^. The screening panel of 204 compounds used in this study covers virtually all FDA-approved small molecule anti-cancer drugs, along with a collection of emerging, investigational and preclinical oncology compounds, including signal transduction inhibitors targeting major oncogenic signaling pathways (Supplementary Table 1). The drugs were plated at 5 concentrations in 10-fold dilution series. The challenge here was to score the individual drug sensitivities in a patient testing setup, where limited sources of fresh, primary cancer cells are available to quantify selective responses in comparison to control cells from healthy donors. Here, we functionally profiled 22 bone marrow aspirates from 14 AML patients, whereas 4 bone marrow samples from healthy donors tested in the same way were used as controls. In the present study, a total of 5,161 sample-compound pairs were analyzed using the DSS analysis pipeline (Figure 1).

**Figure 2:**
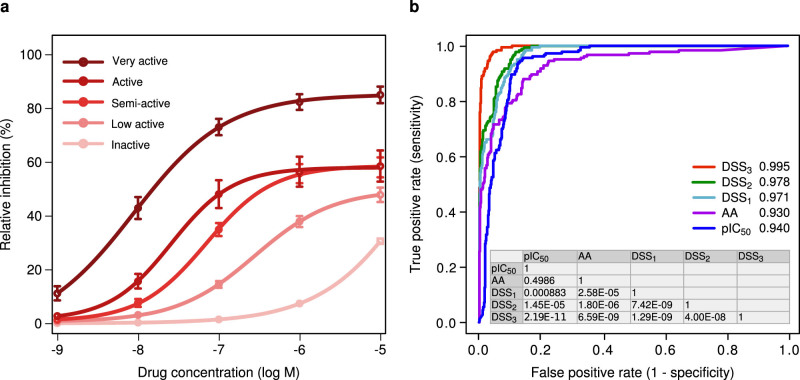
Predictive accuracy of the response scores against a visual evaluation. (a) Average drug-response profiles in five activity classes. The error bars indicate standard error of the mean (SEM). A subset of 795 dose-response curves was visually classified into either inactive (612), low active (70), semi active (65), active (30) or very active (18) classes by an experienced drug screener (T.P.), who was blind to the response parameters during the visual evaluation. The reproducibility of the expert-assigned classifications was confirmed by repeating the visual classification six months later, showing high reproducibility (97.5% of the curves were assigned to the same class by the expert across the five activity classes). (b) Predictive accuracy of each response score was evaluated using the receiver operator characteristic (ROC) analysis, where the dose-response curves were ordered according to the increasing value of the response score (see Methods). The area under the ROC curve (AUROC) is listed for each response score when distinguishing between 612 inactive and 183 active dose-response curves (Supplementary Table 2 details the AUROC for each activity class separately). The statistical significance of observed AUROC differences between the scores (table in the inset) was calculated using the DeLong's test^17^.

To test its quantitative performance, we first systematically evaluated the predictive power of DSS in terms of its accuracy at differentiating between visually-classified active and inactive compounds across the AML patient and control samples (Figure 2a). Especially the integrated and normalized DSS_2_ and DSS_3_ versions systematically improved the sensitivity of the drug efficacy detections at each specificity level, when compared to using the relative IC_50_ parameter alone (*p* < 10^−5^, DeLong's test, DSS vs. relative IC_50_; Figure 2b). The Activity Area (AA) score also showed comparable sensitivity at the highest specificity levels, but its accuracy significantly decreased after moving beyond the most obvious active cases (*p* = 6.6 × 10^−9^, DeLong's test, DSS_3_ vs. AA), resulting in similar overall performance with IC_50_ (*p* = 0.499, DeLong's test, IC_50_ vs. AA; Figure 2b). This was expected since the AA calculation was developed under more focused settings (Supplementary Fig. 1a). While all the response scores could accurately detect the compounds exhibiting the highest efficacy, the DSS_3_ proved especially informative for capturing the subtle differences between the drugs showing low or no activity (Supplementary Table 2).

**Figure 3:**
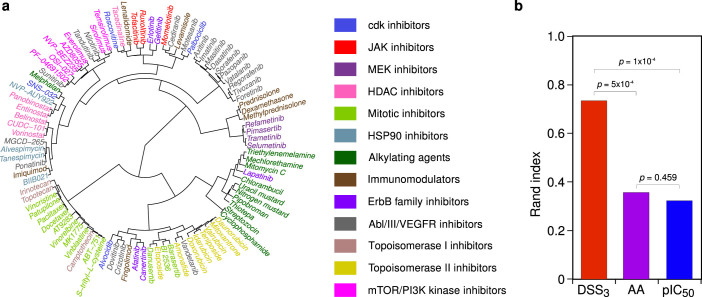
Unsupervised clustering of the compounds based on their drug response profiles. (a) Clustering dendrogram of the compound screening panel. The DSS drug response profiles over all the AML patient samples, relative to the control samples, were clustered using the Ward's hierarchical clustering algorithm^19^ and Spearman's rank-based correlation coefficient (see Supplementary Fig. 6 for dendrograms from the other response scores). The primary mechanism of action (MoA) classification of the compounds is illustrated in color coding (Supplementary Table 1). (b) Comparison of the response scores in terms how accurately their compound clustering reflects the established MoA classes in terms of the adjusted Rand index^21^ (see Supplementary Fig. 2 for other evaluation indices). The empirical statistical significance of the relative differences in the cluster evaluation indices was assessed with respect to permutation-based random null distribution (see Methods).

We next evaluated the performance of DSS in terms of how accurately it can cluster drugs in our oncology compound collection according to their known mechanisms of action (MoA). The differential dDSS response profiles across the AML patient and healthy bone marrow control samples were clustered to reveal similarities and differences in selective drug response patterns between the AML patients (Figure 1e). The unsupervised drug clustering reflected closely the classification of the drugs based on their established MoA (Figure 3a). We note that there is no unambiguous one-to-one mapping between generic MoA classes and many polypharmacological compounds, explaining why the response-driven clustering does not perfectly agree with the MoA-based drug classification. For instance, while majority of VEGFR family and ABL tyrosine kinase inhibitors clustered together, nilotinib and tandutinib clustered with mTOR/PI3K inhibitors. However, DSS_3_ response profiling systematically improved the match to the primary MoA classes, compared with IC_50_ or AA (*p* < 5 × 10^−4^, permutation test; Figure 3b; Supplementary Fig. 2), demonstrating that DSS calculation enables functional grouping of diverse set of compounds in order to predict MoA of uncharacterized drugs.

### DSS calculation improves the response scoring resolution in cancer cell models

**Figure 4:**
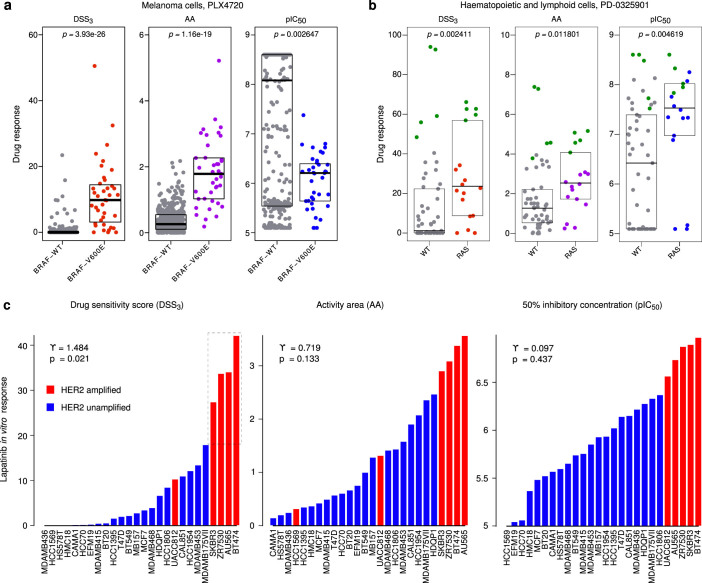
Distributions of the drug response scores in the CCLE *in vitro* cancer cell models. Distribution of the three scores in response to (a) PLX4720 treatment in melanoma cell lines; and (b) PD−0325901 treatment in hematopoietic and lymphoid cells. The boxes depict the median and the interquartile range of the response score, and the *p*-values the difference in the treatment sensitivity between the BRAF-V600E or RAS-mutated and the wild type cells, respectively (Wilcoxon rank-sum test). (c) Individual breast cancer cell responses to lapatinib treatment. The sub-group of highly responsive samples (dotted box) was identified automatically using the observed skewness value *γ* and its significance level (D'Agostino test^15^).

To evaluate how the DSS performs in more controlled settings, we utilized *in vitro* profiling data from the published CCLE study, where various cancer cell lines were screened against 24 anticancer compounds^3^. We first compared the response patterns of PLX4720, a selective RAF family kinase inhibitor, across the wild type BRAF and BRAF-V600E mutated melanoma cell lines; this case study was also used in the original work to demonstrate the operation of the AA score^3^. The AA and DSS_3_ calculations provided similar power to detect the selective sensitivity of PLX4720 treatment in the BRAF-V600E mutated cells, compared with the non-mutated BRAF cells (*p* < 10^−15^, Wilcoxon rank-sum test; Figure 4a, Supplementary Fig. 3). While these two response scores showed similar distributional patterns in the mutated cells, the DSS_3_ had lower responses in the WT cells, compared to the AA, after re-scaling these metrics to the same range (*p* < 10^−50^, Wilcoxon rank-sum test). The IC_50_ response parameter was not able to detect the selectivity of PLX4720 to BRAF-V600E mutated cells, rather it scored wild-type cells as more sensitive to PLX4720. These results indicate that the DSS outperforms the conventional activity metrics, such as IC_50_, and shows comparable selectivity to that of the recently introduced AA measure in this selected cell line case study.

In the second CCLE case example, we compared the distributions of the three activity scores in response to the MEK1/2 kinase inhibitor PD−0325901 in hematopoietic and lymphoid cell lines with or without RAS mutations based on the fact that the MEK1 and MEK2 kinases are key signaling components downstream of RAS oncogenes. Similar to the BRAF-V600E:PLX4720 example, DSS_3_ and AA were able to detect that the RAS-mutated cell lines as a group were more sensitive to MEK1/2 inhibition compared with non-mutated cells (*p* = 0.0024 and *p* = 0.012, respectively, Wilcoxon rank-sum test; Figure 4b). Interestingly, among the most MEK inhibitor sensitive cell lines, there were also several non-RAS mutated cells, indicating that the RAS mutational status is not the sole determinant of the MEK inhibitor sensitivity. Upon closer inspection of these highly sensitive cells (highlighted in green in Figure 4b and Supplementary Table 3), we noticed that they were predominantly AML-derived cell lines, irrespective of their mutational status. Hence, while MEK inhibitors are not magic bullets for treating cancers carrying mutated RAS isoforms in general, a sub-population of AMLs, including those with RAS mutations, appear highly addicted to MEK signaling and might serve as a promising disease cohort to explore MEK inhibitor therapy.

As a third application case, we selected data for a set of 26 breast cancer cell lines from the CCLE resource and studied their differential responses to lapatinib, a clinically approved dual EGFR and ERBB2 (HER2) kinase inhibitor. A subset of four cell lines resulted in significantly higher DSS_3_ response, compared with the others (*p* = 0.00013, Wilcoxon rank-sum test), suggesting that these lines are addicted to HER2 signaling (Figure 4c). Such multimodality was not seen in either the IC_50_ or AA distributions (Figure 4c), whereas the sensitized sub-group was readily detectable by significant positive skewness in the DSS_3_ distribution (*γ* = 1.484, *p* = 0.021, D'Agostino test). All the four cell lines (SK-BR-3, ZR-75-30, AU-565, and BT-474) are known to harbor HER2 amplifications and overexpression. To confirm that these responses were specifically linked to HER2 addiction, we showed that the four lapatinib-responsive cell lines were insensitive to erlotinib (*p* > 0.75, Wilcoxon rank-sum test), a compound that is known to target specifically EGFR but not HER2 (Supplementary Table 4). Interestingly, in the set of 26 breast cancer cells, there were also HER2-amplified cases, such as HCC1569, which showed relatively low sensitivity to lapatinib (Figure 4c), indicating that HER2 positivity does not necessarily imply HER2 addiction.

### DSS calculation improves the identification of drug-sensitive AML patient groups

**Figure 5:**
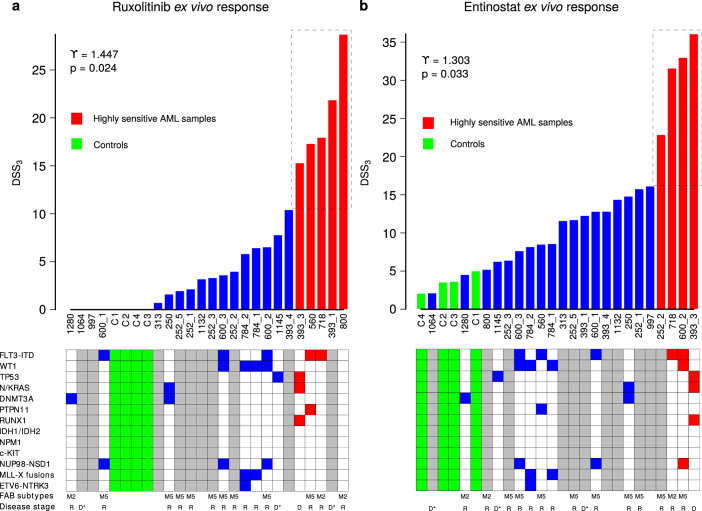
Distributions of DSS_3_ responses across the primary cancer samples *ex vivo*. The AML patient and control sample responses to (a) ruxolitinib and (b) entinostat. The sub-group of highly responsive samples (dotted box) was identified automatically using the observed skewness values *γ* and their significance levels (D'Agostino test^15^). Tables below list the molecular profiles (significant AML mutations and recurrent gene fusions), disease stages (D, diagnosis; D*, secondary AML diagnosis; R, relapsed and/or refractory), and French–American–British (FAB) classification of the patients to illustrate the lack of correlation between functional drug sensitivity and somatic mutation profiles in this limited cohort. Examples of drug-response curves behind some of the individual response values are shown in Supplementary Fig. 7.

Finally, we identified a number of examples of translational importance, where positive skewness of the DSS distribution allowed us to distinguish sub-groups of AML patient samples with unique and novel sensitivities to specific drugs. The first such example is ruxolitinib, a recently approved JAK inhibitor for myelofibrosis, which showed increased differential DSS_3_ response in five AML patient samples (*p* = 0.00063, Wilcoxon rank-sum test; Figure 5a), but whose selective response was totally missed by the AA or IC_50_ distributions (Supplementary Fig. 4). Ruxolitinib has previously been explored in patients with relapsed or refractory leukemias, with results showing high heterogeneity in individual response patterns^10^. It is currently undergoing phase II trials for advanced adult AML patients (clinicaltrials.gov; NCT01251965; NCT00674479), but surprisingly without any molecular or functional biomarkers as inclusion criteria, suggesting that the response rate may end up being low. Strikingly, the DSS-based sample stratification gave us novel insights into the characteristics of those advanced AML cases that are highly responsive to JAK inhibitors *ex vivo*, which could be promising cases to treat with ruxolitinib in the clinic.

Similarly, we observed that *ex vivo* drug response to the histone deacetylase (HDAC) inhibitor entinostat resulted in distinctly multimodal DSS_3_ distribution, where all of the controls and most of the patient samples were clustered into a low-response background group, whereas four of the patient samples formed an distinct outlier group (252_2, 718, 600_2, and 393_3), which showed significantly higher response levels (*p* = 0.00013, Wilcoxon rank-sum test; Figure 5b; Supplementary Fig. 4). Entinostat is currently undergoing several phase II clinical trials for treatment of various cancers, including AML and myelodysplastic syndrome. Also in this case it is striking that no molecular or phenotypic biomarker inclusion criteria are used in the ongoing trials. Based on our results, entinostat may induce beneficial epigenetic modifications in a specific subgroup of AML patients only, warranting its further testing in more stratified clinical trials. In general, these examples demonstrate that DSS calculation provides a quantitative and highly selective means to identify drug-sensitive subgroups of patient samples that are likely to benefit from a particular drug treatment.

**Figure 6:**
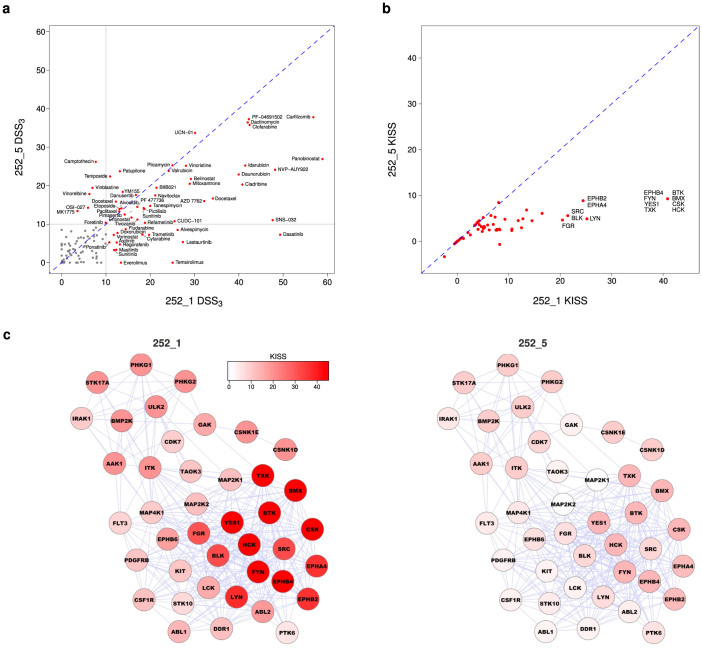
Monitoring of treatment response using drug sensitivity and target addiction profiling. (a) Correlation of DSS_3_ response profiles in an individual patient (252) before and after dasatinib treatment (252_1 vs 252_5). (b) Correlation of target addiction profiles estimated with the kinase inhibition sensitivity score (KISS, see Methods). (c) Network view of the kinase addiction changes before and after treatment (left and right panels, respectively). The kinase addiction sub-networks show connections among the initially most active and selective kinase targets (KISS > 5 in the sample 252_1). Node coloring indicates the degree of kinase addiction (KISS, Eq. (2)), and edges connect kinases with similar inhibitor selectivity profiles (Spearman's rank-based correlation > 0.5) based on a biochemical screen of kinase inhibitor specificities^11^. Non-expressed kinase targets were excluded from the networks. Dynamic changes in the kinase addiction maps during the all the serially sampled phases of the disease progression in this patient are shown in Supplementary Fig. 8.

A majority of AML patient samples *ex vivo* appear to be addicted to kinase signaling^5^,^6^. To map the molecular dependencies in the patient cells, we compared the dDSS response profiles with the drug target profiles from a published set of kinase inhibitor specificities^11^. This allowed us to identify potential kinase-driven signals that the particular patient cells may be addicted to (see Methods). As a case study, we studied two serial samples from the same AML patient (252) before and after treatment with the tyrosine kinase inhibitor dasatinib. At the compound-level, DSS highlighted a reduced sensitivity to a number of kinase inhibitors, such as dasatinib, after the treatment (Figure 6a). At the target-level, the kinase addiction score supported the decreased addiction to the activity of multiple kinase targets (Figure 6b). The kinase addiction network provided an additional view of changes in the target addiction scores before and after the dasatinib treatment (Figure 6c). Such integrated network approach facilitates not only mapping of the key oncologic signals underlying the initial treatment sensitivity, but also following-up and understanding the mechanisms behind the acquired resistance during the disease evolution^6^.

## Discussion

We have shown that the model-based drug sensitivity quantitation effectively captures and integrates complementary information extracted by IC_50_, slope and other activity parameters from the complex dose-response relationships. The importance of considering information from multiple response parameters was recently shown in cancer cell line drug testing applications^9^. We used here AML as the primary disease model since the driving molecular signals underlying AML are still poorly understood, and there is no standardized and effective second line AML treatment, resulting in very poor prognosis for relapsed patients. While next-generation sequencing of clinical AML samples has allowed for extensive cataloging of recurring mutations, these have not yet provided links to clinically actionable therapeutic strategies in most individual cases, perhaps because of our limited understanding of the complex genetic events and extensive clonal heterogeneity that induce and drive an AML^6^,^12^. The difficulty of making predictive links between the molecular patterns and drug sensitivity or resistance was also exemplified here in the two AML patient examples (Figure 5). The DSS calculation was implemented and tested in this study to provide a standardized means for functional investigation of druggable vulnerabilities in individual cancer samples *ex vivo*, even in the absence of genetic or epigenetic profiling information, thereby providing complementary insights into cancer phenotypes and cellular addictions on an individualized basis.

The continuous model estimation makes the DSS calculation robust against many sources of technical variability. For instance, the model fitting enables interpolation of missing values at intermediate concentration levels. Further, while many of the single response parameters, such as IC_50_, are dependent on the concentration ranges being tested, the summary response metrics, such AA and DSS, provide more comparable results also for compounds tested under different concentration windows. The robustness of the area-based metrics was confirmed in AML cell line models, where AA and DSS response profiles showed improved reproducibility compared to IC_50_ (Supplementary Fig. 5). Increased inconsistency and problems in extrapolating IC_50_ levels was also recently noted in the comparison between CCLE and Sanger cell line drug testing data^13^. Importantly, area-based metrics enable straightforward calculation of the differential responses, relative to that of the control samples, while differential IC_50_ is not so straightforward to interpret. In many case examples, the waterfall plots of the drug response distributions over a set of patients were relatively uniform when plotted using standard metrics such as IC_50_. In contrast, especially the DSS_3_ version, which involved further normalization of AUC by the active dose range, was shown to amplify the differential responses, making the systematic identification of sensitive patient subgroups more straightforward (e.g. Figures 4 and 5).

The previously introduced AA metric provides an approximation of the AUC through a discrete rectangle method (Supplementary Fig. 1a). AA was shown to work well on a smaller collection of 24 compounds, with relatively narrow bioactivity spectra and densely-sampled concentration ranges, carefully centered around an expected IC_50_ for the primary target of the inhibitors to avoid off-target effects^3^. However, such discrete approximation may provide sub-optimal response estimates under other settings, especially when broader bioactivity spectra are being tested with a more complex set of compounds. The basic version of DSS (named DSS_1_) is an AUC measurement with the baseline noise subtracted and therefore conceptually similar to AA (Supplementary Fig. 1b). However, the further normalizations of the DSS calculation make the DSS_2_ and DSS_3_ versions different from the AA. In particular, DSS_3_ captures additional dose-response relationships, and it was shown to outperform AA in the AML case studies, which rely on sparsely-sampled data from limited sources of primary cells (Figure 2 and 3, Supplementary Figs. 2 and 4). An additional advantage of DSS_3_ in the clinical settings is its reduced correlation with the blast counts (Supplementary Fig. 9), which make samples with varying leukemic blast percentages easier to cross-compare. Further, DSS_3_ version is able to distinguish toxic response patterns that show activity at the highest dose levels only from the clinically more relevant patterns that show potency over a wider therapeutic window, even if their AUC is similar (see e.g. the entinostat example in Supplementary Fig. 7).

The adequate performance of the DSS was shown here both in controlled cancer cell line models as well as in clinical patient-derived applications. Therefore, we believe it should benefit a range of drug testing applications *in vitro* and *ex vivo*. Further, it was shown in our previous work that the observed *ex vivo* drug responses are predictive of the *in vivo* treatment response observed in the clinic^6^. As with any response score, however, the dose-response curves behind the top DSS hits should be visually confirmed before clinical decision making. As a future development, calculation of confidence intervals would provide estimate of the uncertainty of response scores, such as IC_50_, AUC and DSS, in studies where enough sample material is available for technical replicates or increased number of dose levels sampled for parameter confidence estimation. However, the current DSS implementation was already shown to enable statistical identification of such patient subgroups that are most likely to benefit from a treatment (Figures 4 and 5), whereas DSS profiles across the compounds revealed mechanistic similarities among those drugs showing correlated response patterns (Figure 3). Further, when combined with information on the cellular targets of the most sensitive and selective drugs, one cannot only start identifying pharmacologically targetable oncogenic driver signals (Figure 6), but also to monitor and identify potential mechanisms behind *in vivo* emerging resistance to the targeted agents^6^. We expect that this integrated approach will help us to predict next line of more effective treatment strategies, such as multi-targeted combination therapies^14^, for each individual refractory patient, and will complement the genomic profiling approaches for AML and other cancers.

## Methods

### Patient material

As a primary clinical evaluation material, we used a set of 22 bone marrow aspirates from 14 mainly relapsed and refractory AML patients, as well as 4 bone marrow samples from healthy donors as controls from our ongoing study^6^. All the samples were fresh and collected in EDTA treated tubes after informed consent with approval (No. 239/13/03/00/2010, 303/13/03/01/2011), in accordance with the ethical standards of the Helsinki University Central Hospital (HUCH), approved by HUCH Institutional Review Board (Dnro 60/2011). The drug sensitivity and resistance testing (DSRT) was performed as previously described^6^. Briefly, *ex vivo* DSRT was run on mononuclear cells isolated from AML patient or healthy bone marrow aspirates using Ficoll density gradient (Ficoll-Paque PREMIUM; GE Healthcare) suspended in Mononuclear Cell Medium (MCM; PromoCell) supplemented with 0.5 μg/mL gentamicin and 2.5 μg/mL amphotericin B. The oncology screening panel used in the present study included 204 compounds covering the approved cancer small molecule pharmacopeia and the active substances of emerging investigational and experimental anticancer compounds, including signal transduction inhibitors targeting major kinase and non-kinase targets (Supplementary Table 1). The compounds were dissolved in DMSO and pre-printed on tissue culture treated 384-well plates (Corning) with an acoustic liquid handling device, Echo 550 (Labcyte Inc.). Each compound was tested in five different concentrations covering a 10,000-fold concentration range (no technical replicates). Prior to addition of the cells, the compounds were dissolved in 5 μL of MCM for 30 min on a plate shaker. Single cell suspension (10,000 cells/well in 20 μL) was transferred to each well with a peristaltic dispenser (MultiDrop Combi; Thermo Scientific). The plates were incubated at 37°C for 72 h, after which the cell viability was measured using CellTiter-Glo luminescent assay (Promega) according to manufacturer's instructions with a Molecular Devices Paradigm plate reader. The response readout was normalized in relation to negative control (DMSO) and positive control (100 μM benzethonium chloride), resulting in relative growth inhibition %. The raw dose-response data were processed in Dotmatics Browser/Studies software (Dotmatics Ltd.), and then subjected to the DSS analysis pipeline (see Supplementary Methods).

### Cell line material

As an additional evaluation material, we made use of the set of 479 cell lines screened against 24 anticancer compounds from the *Cancer Cell Line Encyclopedia* (CCLE) resource^3^. Dose response for those compounds was measured in dilutions at 8 different concentrations, namely 2.5, 8, 25, 80, 250, 800, 2,530 and 8,000 nM. The medians over the technical replicates of the dose responses were used in the DSS calculation (Supplementary Methods). In the CCLE cell line case studies, we used the Activity Area (AA) values reported in the Supplementary material of the original work^3^. For comparative evaluations in our AML samples and controls, we implemented the Activity Area calculation according to the descriptions in the Supplementary Material of the original work^3^, with the help of instructions from one of the authors (personal communication with Dr. Joseph Lehár). The AA calculation is based on discrete summing of the differences between the measured response (relative growth inhibition %) and the reference level (response set to zero) over the eight dose levels (so-called rectangle approximation of the integral function, see Supplemental Fig. 1a). AA has a value of zero, when there is no drug activity and eight for compounds with 100% inhibition across all the eight drug concentrations.

### Statistical analysis

To objectively identify compounds whose response distributions show exceptional positive response, that is, a relatively few highly responsive samples at the right tail of the drug response distribution, we calculated the sample skewness *γ* of the drugs' empirical response distribution over all the samples under analysis. The one-sided significance *p*-value of the observed positive skewness was assessed using the D'Agostino^15^ test in the R-package “moments” (version 0.13, http://cran.r-project.org/package=moments). This enables systematic detection of drug-sensitive patient sub-groups for a given compound, without visually going through all the drug response distributions. When comparing two sets of samples, such as highly responsive patient samples against the remaining samples for those compounds initially identified with positive skewness, we assessed the difference in the response levels between the two pre-defined sample groups with the Wilcoxon rank-sum test. We chose to use the non-parametric test because the response distributions cannot be assumed to be normally distributed.

The predictive accuracy of the DSS, IC_50_ and AA metrics was assessed in terms of their capability to distinguish the active dose-response curves from the inactive ones using the receiver operating characteristic (ROC) analyses; ROC curves evaluate the relative trade-off between true positive rate (sensitivity) and false positive rate (1 – specificity) of the metric when ordering the dose-response curves according to the increasing value of the response metric^16^. The overall accuracy of each response metric was summarized using the area under the ROC curve (AUROC) measure; for an ideal metric, AUROC = 1, whereas a random metric obtains an AUROC = 0.5 on average. Statistical significance of an observed AUROC, when compared to random classifier, was assessed using the roc.area function in the R-package “verification”. Statistical significance of an observed AUROC difference between two response metrics was assessed using the “pROC” package with the De Long's test^17^.

### Kinase addiction scoring

To identify the selective kinase targets the individual AML samples may be addicted to, we compared the sample-specific dDSS response with the target profiles of 35 kinase inhibitors overlapping between our compound panel and the kinase inhibitors whose target specificity was biochemically profiled in a recent kinome-wide study^11^. We designed a kinase inhibition sensitivity score (KISS), which estimates how sensitive the cells are to inhibition to a specific kinase target (or in other words, the addiction to the activity of the given kinase). Formally, for each kinase target *k*, we calculated KISS by summing the dDSS values over those kinase inhibitors *i* that selectively target *k*: 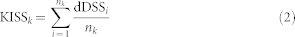
Here, the sum is through those *n_k_* inhibitors that specifically target the kinase *k* and whose skewness *γ* shows significant positive selectivity (*p* < 0.05, D'Agostino test^15^). These selective drug response and target profiles were used to define putative kinase addiction pathways for each individual sample, that is, the connected sets of selective kinases that the individual leukemia cells are likely to be addicted to. This is similar to the concept of kinase pathway dependence^5^. The identified kinase addiction sub-networks for the patient samples were visualized using the automated layout options in the Cytoscape network analysis software^18^.

### Response profile clustering

To reveal similarities and differences in the drug response patterns over the samples, the DSS, IC_50_ and AA drug response profiles were grouped into functionally similar drug clusters using unsupervised hierarchical clustering technique, Ward's algorithm^19^. The Spearman's correlation coefficient was used as the similarity function, because the rank-based correlation provided relatively robust and reproducible results between different runs. The evaluation of the clustering solutions was carried out using external cluster evaluation indices, which measure performance by matching the identified clustering solution to a priori information of the drugs. Here, the external benchmarking drug clusters corresponded to the known mode of action (MoA) classes of the drugs, if available (Supplemental Table 1). MoA classes with less than three drugs were excluded, since these present too narrow and potentially unstable drug classes for the cluster validation purposes.

More specifically, we first determined the response score-based drug clusters by cutting branches off the hierarchical clustering dendrogram using the “dynamicTreeCut” library^20^. The obtained drug partitions were then compared to the MoA drug classes using thee different cluster evaluation indices. The Rand index has a value between 0 and 1, with 0 indicating that the two partitions do not agree on any pair of drugs, and 1 indicating that the drug clusters are exactly the same^21^. In the Jaccard index, value 1 indicates that one of the partitions lies completely within the other, and value 0 indicates that the partitions have no common drugs. With the Fowlkes–Mallows index, a higher value indicates a higher similarity between the two drug partitions, whereas for two unrelated partitions the index approaches zero as the number of drugs increases^22^.

Statistical differences in the cluster evaluation indices between the response scores were tested through permutation-based null-distributions. More specifically, a large set of random cluster assignments was simulated by randomly shuffling the drug labels in the observed clustering solutions, separately for DSS, IC_50_ and AA metrics, while preserving the number of clusters in the original clustering solutions. The random null-model for the differences was obtained by taking pairwise differences in the index values between any two random drug cluster assignments (e.g one for DSS and the other for AA score). The empirical *p*-value was calculated by counting the number of the pairwise random permutations having greater or equal index difference value when compared to the observed difference, divided by the number of random permutations (here: 10,000).

## Author Contributions

B.Y., T.P., K.W. and T.A. developed and implemented the DSS calculation. A.S., O.K., K.W. and T.A. developed and implemented the KISS calculation. T.P., E.K. and K.W. participated in the development of DSRT infrastructure. M.K. provided and coordinated sampling of patient material. R.K., M.M.M., D.M. and A.M. performed sample and cell preparation. B.Y., T.P., J.K., K.P., C.H., O.K., K.W. and T.A. designed the study, supervised the experimental, clinical and computational analysis and wrote the manuscript.

## Supplementary Material

Supplementary Information Supplementary Information
