# Supplementary material for: Quantitative scoring of differential drug sensitivity for individually optimized anticancer therapies
Source: Sci Rep. 2014 Jun 5;4:5193. doi: 10.1038/srep05193 (PMC4046135; doi:10.1038/srep05193)
Supplement: Supplementary Information [file srep05193-s1.pdf]

SUPPLEMENTARY METHODS FOR

Quantitative scoring of differential drug sensitivity for individually optimized anticancer therapies

Bhagwan Yadav, Tea Pemovska, Agnieszka Szwajda, Evgeny Kulesskiy, Mika Kontro, Riikka Karjalainen, Muntasir Mamun Majumder, Disha Malani, Astrid Murumägi, Jonathan Knowles, Kimmo Porkka, Caroline Heckman, Olli Kallioniemi, Krister Wennerberg and Tero Aittokallio

Drug sensitivity scoring pipeline

The drug sensitivity scoring (DSS) pipeline takes as its input individual dose-response values, which are first modeled using appropriate fitting functions, such as logistic model, and then scored using analytic continuous integration; the outputs of the pipeline include, for instance, waterfall plots of the individual drug selectivities, heatmap plots of the DSS profiles over all the samples, as well as network maps of the addicted kinases underlying the individual response profile (Figure 1). The next sections detail the DSS analysis pipeline and its implementation.

Logistic curve fitting

Let us denote the observed dose-response data obtained from a drug screening experiment as a vector of cell viability readout values (mean or median values, if replicates available) over the selected concentration range ($y_{C_{\min}}$,..., $y_{C_{\max}}$). To model the underlying response function y as a continuous function of the variable dose x, the observed drug response data is modeled using a non-linear dose-response function. By default, the 4-parameter logistic function is used here:

$$y = d + \frac{a-d}{1+10^{b(c-x)}}. \qquad \text{(Eq. 1)}$$

Here, *a* is the maximal response (i.e., the top asymptote of the curve), *b* is the slope of the curve, *c* is the IC_{50} (i.e., half-maximal inhibitory concentration), and *d* is minimal response (i.e., the bottom asymptote of the curve). These correspond to the symbols R_{max}, Slope, IC_{50} and R_{min} used in the text. We note that the other commonly used alternatives, such as the sigmoidal or Hill slope functions, provide similar functional forms, and could be used instead.

To calculate the DSS response values for individual dose-response vectors, the logistic curve fitting in the AML primary and control samples was carried out using the Dotmatics Ltd. Browser/Studies software suite (http://www.dotmatics.com/products/studies/), while in the CCLE cell line material[1] the logistic modeling of the original dose-response data was carried out using the GraphPad Prism software package (http://graphpad.com/prism/Prism.htm).

DSS derivation and calculation

The *area under the curve* (AUC) is the area covered between the dose-response curve (Eq. 1) and the concentration *x*-axis. For analytic calculation of the AUC, we derived a closed-form exact solution for the definite integral of *y* over the selected concentration range from x_1 to x_2:

$$\mathrm{AUC} = \int_{x1}^{x2} y\,(x)\mathrm{d}x = Y(x_2) - Y(x_1), \qquad (\mathrm{Eq.}\,2)$$

where the integral function of the dose-response can be analytically expressed as

$$Y(x) = \frac{(a-d)\log_{10}\left(1+10^{b(c-x)}\right)}{b} + ax. \qquad (\mathrm{Eq.}\,3)$$

By default, we start the integration from the concentration at which the drug-response curve crosses the minimum activity level t (by default, $t = 10\%$, Supplemental Figure 1B; corresponds to A_{min} in Figure 1C):

$$x_1 = c - \frac{\log_{10}(a-t) - \log_{10}(t-d)}{b}. \qquad (\mathrm{Eq.}\,4)$$

In many high-throughput screening applications, the minimum response level is set to zero for each drug (i.e. $d = 0$). In this special case, using Eqs. 2-4, the AUC takes the following analytic form:

$$\mathrm{AUC} = a\left[x_2 - c + \frac{\log_{10}\left(1+10^{b(c-x_2)}\right) + \log_{10}\left(1-\frac{t}{a}\right)}{b}\right].$$

After subtracting from the integrated total AUC, the area below the minimum activity level, and then dividing the remaining difference by the maximal response area, we get the basic version of the *drug sensitivity score* (DSS), which is effectively a normalized version of standard AUC:

$$\mathrm{DSS}_1 = \frac{\mathrm{AUC} - t(x_2 - x_1)}{(100-t)(C_{\mathrm{max}} - C_{\mathrm{min}})}.$$

Here, C_{min} and C_{max} are the minimum and maximum concentrations, respectively, at which the drug was screened (in a typical AML screen, for instance, $C_{\mathrm{min}} = 1$ nM and $C_{\mathrm{max}} = 10{,}000$ nM, and in the CCLE screens, $C_{\mathrm{min}} = 2.5$ nM and $C_{\mathrm{max}} = 8000$ nM).

To normalize the effect of maximal response at the highest drug concentration, especially when $x_2 = C_{\max}$ (default option), which many times corresponds to off-target toxicity, the DSS was further divided by the logarithm of the top asymptote a:

$$\mathrm{DSS}_2 = \frac{\mathrm{DSS}_1}{\log_{10} a}.$$

To further emphasize those drugs that obtain their response area over a relatively wide dose window, as compared to drugs that show increased response only at the higher end of the concentration range, we further modified the score:

$$\mathrm{DSS}_3 = \mathrm{DSS}_2 \frac{x_2 - x_1}{C_{\max} - C_{\min}}.$$

We set DSS=0 in those cases where the estimated IC50 (parameter c) is at or beyond the maximum dose level tested $C_{\max}$, since such drug responses are often associated with off target effects that most likely are clinically irrelevant.

With each version of the DSS-score, the *differential drug sensitivity score* (dDSS) is calculated by subtracting the average of the control DSSs from the patient DSS, in case one or several control samples are available (e.g. the healthy bone marrow samples in the AML application).

Ranking of putative addicted kinases

To identify selective kinase targets the individual AML samples may be addicted to, we compared the sample-specific dDSS response with the target profiles of 35 kinase inhibitors overlapping between our compound panel and the compounds whose specificity was biochemically profiled in a recent kinome-wide profiling study[2]. More specifically, for each kinase target *k*, we calculated *Kinase Inhibition Sensitivity Score* (KISS) by summing the dDSS values over those kinase inhibitors *i* that selectively target *k*:

$$\mathrm{KISS}_k = \sum_i \frac{\mathrm{dDSS}_i}{n_k}.$$

Here, *i* goes through all those kinase inhibitors that specifically target the kinase *k* and whose skewness γ shows significant positive selectivity ($p < 0.05$); n_k is the number of kinase inhibitors shown to target the kinase *k* on the basis of the biochemical kinase inhibitor specificities[2]. These selective drug sensitivities were used to define a putative "*kinaddictome*" for each patient sample - the kinases that the individual leukemia cells are likely to be addicted to, and therefore could provide important therapeutically actionable targets. The kinase sub-networks for the samples were visualized using the Cytoscape network analysis software[3].

Data clustering

To reveal similarities and differences in selective drug response over the samples, differential DSS response profiles for individual drugs and samples were grouped into functionally similar drug clusters using unsupervised hierarchical clustering technique, Ward's algorithm[4]. We used here the Spearman correlation coefficients as the similarity function in the clustering algorithm, because the rank-based correlation provided relatively robust and reproducible results between different runs. The evaluation of the activity score-based clustering results was carried out using external cluster evaluation procedures, where the external benchmark drug clusters correspond to the known mode of action (MoA) classes of the drugs, if available (Supplemental Table 1). More specifically, we first determined the DSS-based drug clusters by cutting branches off the hierarchical clustering dendrogram using the "dynamicTreeCut" library[5]. These drug partitions were then compared to the MoA drug classes, excluding MoA classes with less than three drugs.

Cluster evaluation

To measure the similarity between the established MoA classes and the DSS-based drug partitions, we used adjusted version of *Rand index*, where the expected agreement between two random partitions is calculated by means of the generalized hypergeometric distribution[6]. The adjusted Rand index lies between 0 and 1, where 0 indicates random drug clustering and 1 that the two partitions agree perfectly. The adjusted Rand index was calculated using the R-package "fossil"[7]. We further validated the hierarchical clustering results using two additional cluster evaluation measures. The *Jaccard index* measures the similarity between the two drug partitions by calculating the size of their intersection divided by the size of the union of the two drug partitions. An index of 1 means that one of the partitions lies completely within the other, and an index of 0 indicates that the datasets have no common drugs. With the *Fowlkes–Mallows index*, a higher value indicates a greater similarity between the two drug partitions, and for two unrelated partitions the index approaches zero as the number of drugs increases[8].

Implementation

The DSS calculation and data analysis pipeline was implemented in R programming language (version R-2.14.1, http://www.r-project.org/). In addition to the specific R-packages mentioned above, the following packages or libraries were used in the implementation: "xlsx" for reading and writing Excel documents, "stringr" for handling character strings, "gplots" for plotting heatmaps and histograms, "beanplot" for plotting density plots, "hopach" for calculating distances between samples and drugs, "pROC", "ROCR", "caTools" and "verification" for the ROC analysis. The R-package and its source code implementing the DSS calculations are freely available at Google code website (https://dss-calculation.googlecode.com/svn/trunk/).

References

1. Barretina, J. *et al.* The Cancer Cell Line Encyclopedia enables predictive modelling of anticancer drug sensitivity. *Nature* **483**, 603–7 (2012).
2. Davis, M. I. *et al.* Comprehensive analysis of kinase inhibitor selectivity. *Nature Biotechnology* **29**, 1046–51 (2011).
3. Shannon, P. *et al.* Cytoscape: a software environment for integrated models of biomolecular interaction networks. *Genome Research* **13**, 2498–504 (2003).
4. Ward, J. H. Hierarchical grouping to optimize an objective function. *Journal of the American Statistical Association* **58**, 236 (1963).
5. Langfelder, P., Zhang, B. & Horvath, S. Defining clusters from a hierarchical cluster tree: the Dynamic Tree Cut package for R. *Bioinformatics* **24**, 719–20 (2008).
6. Hubert, L. & Arabie, P. Comparing partitions. *Journal of Classification* **2**, 193–218 (1985).
7. Vavrek, M. J. fossil: palaeoecological and palaeogeographical analysis tools. *Palaeontologia Electronica* **14**, 1T (2011).
8. Fowlkes, E. B. & Mallows, C. L. A method for comparing two hierarchical clusterings. *Journal of the American Statistical Association* **78**, 553 (1983).

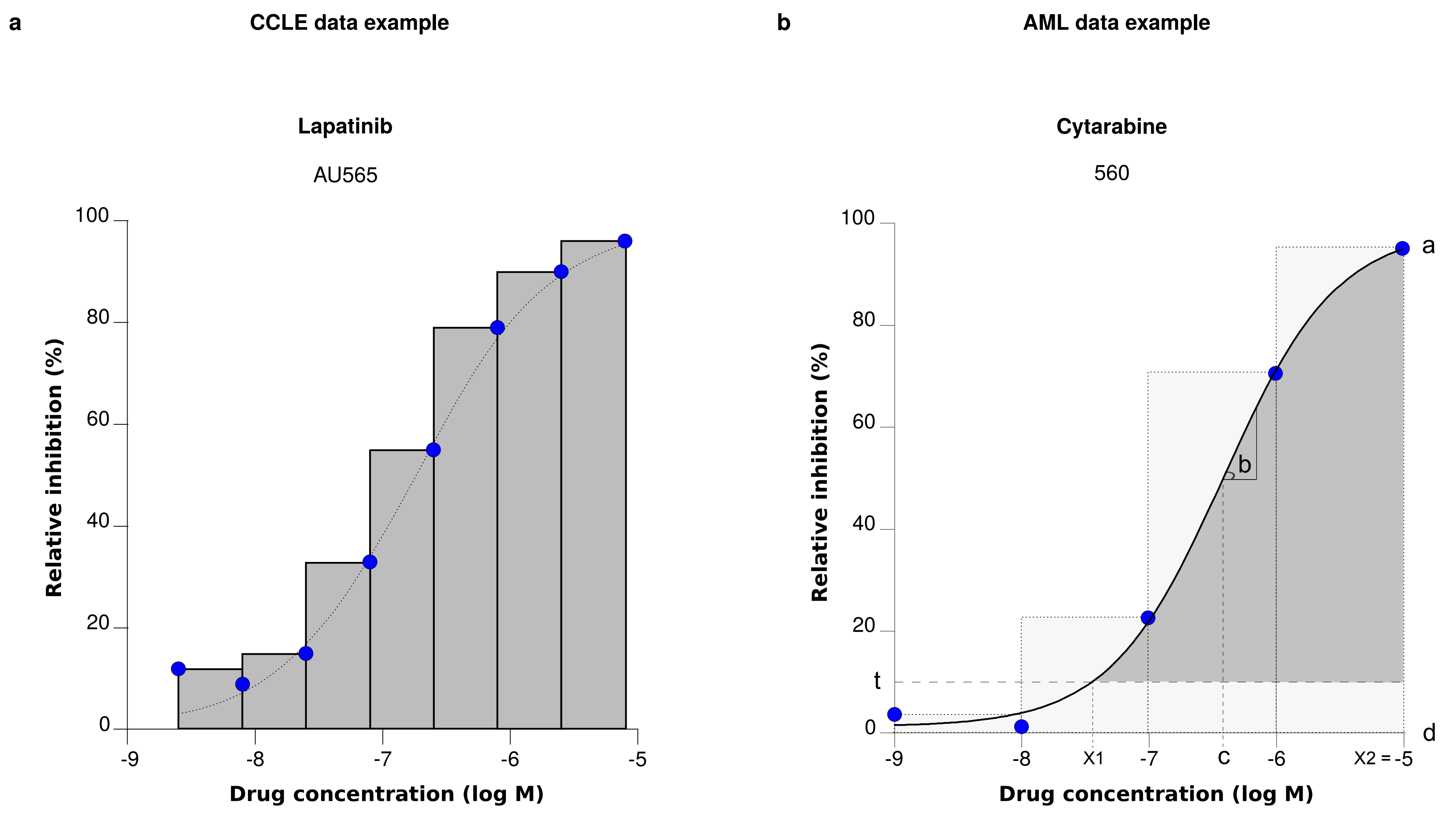

Supplementary Figure 1. (A) An example of the activity area (AA) calculation in a CCLE breast cancer cell line. AA corresponds to the so-called rectangle method, discrete approximation of the integral of the continuous dose-response function (dotted curve), made by summing the area of a collection of rectangles (the grey area), whose heights equal to the values of the observed responses at pre-defined concentration points (8 dose levels in the CCLE data). As the number of dose levels gets larger, this approximation becomes more accurate. (B) An example of the drug sensitivity score (DSS) calculation in an AML patient sample. DSS uses a continuous model (here logistic function, solid curve) to compute the integral of the dose-response function (the grey area). Such interpolation-based method improves the accuracy of the response estimates, especially in settings where relatively wide concentration range is sparsely sampled (5 dose levels in our screening setup). In such a setup, the rectangle method (dotted histogram) may result in sub-optimal response estimates. To avoid summing up insignificant activities, the integration is performed over those concentrations where the response goes beyond the pre-defined minimum activity level (symbol t, here set to 10%). The symbols a, b, c, d and t refer to the mathematical equations in Supplementary Material, and correspond to the symbols R_{max}, Slope, IC_{50}, R_{min}, and A_{min} used in the text and in Figure 1c.

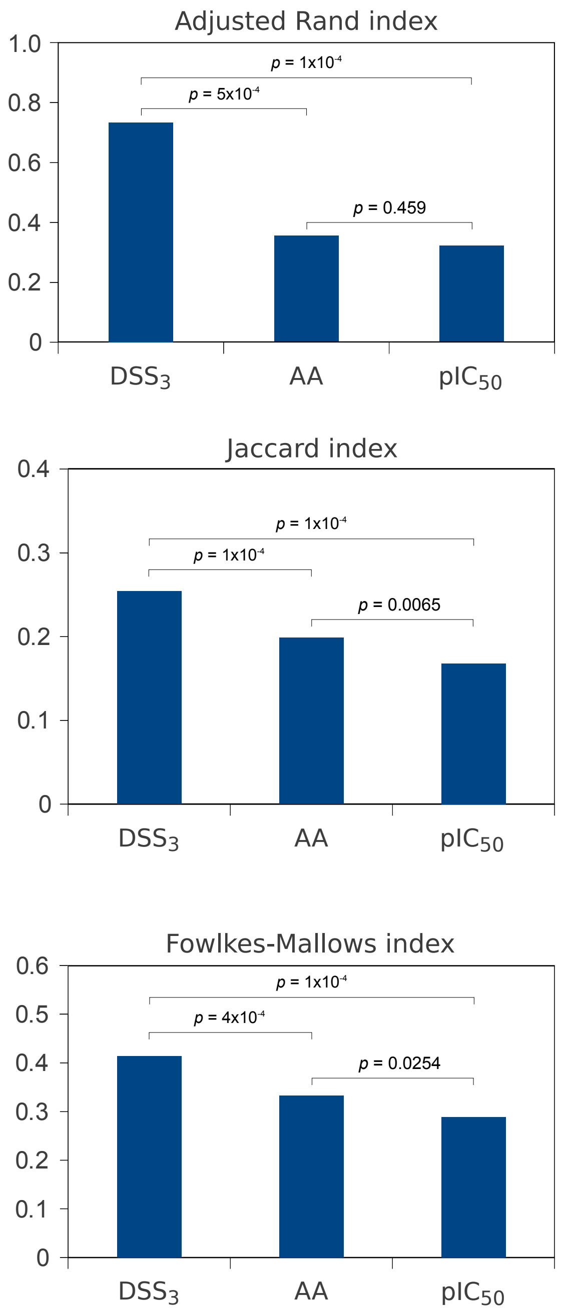

Supplementary Figure 2. Three external cluster validation indices applied to evaluate the drug classifications made using the response profiles from the different activity scores (DSS_3, AA and pIC_{50}), together with the Ward's unsupervised hierarchical clustering and Spearman's rank-based correlation metric. With each index, a higher value indicates a greater similarity between the activity score-based clustering and the established mode of action classification of the drugs, and the zero base line indicates random or non-overlapping drug clustering. The number of drug clusters detected by cutting branches off the hierarchical clustering dendrogram using the dynamic tree cut algorithm was 8, 11 and 10 for the DSS_3, AA and pIC_{50} -based classifications, respectively. The differences in the number of clusters was taken into account in the permutation-based statistical testing of the differences (empirical p-values, see Methods), where the random cluster partitions were forced to preserve the same number of clusters than those in the original clustering solutions.

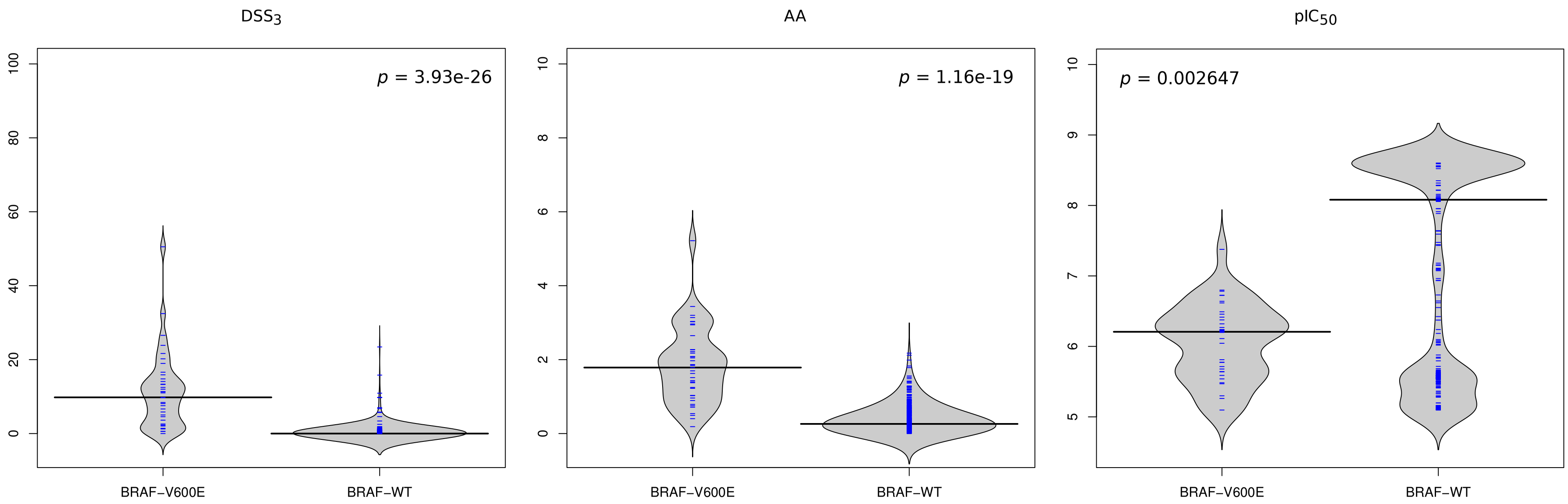

Supplementary Figure 3. Distribution of the three activity scores in response to PLX4720 treatment in melanoma cell lines of the CCLE resource. The solid horizontal lines indicate median response and the p-values the statistical significance of the difference in the treatment sensitivity between the BRAF-V600E mutated and the wild type BRAF cells.

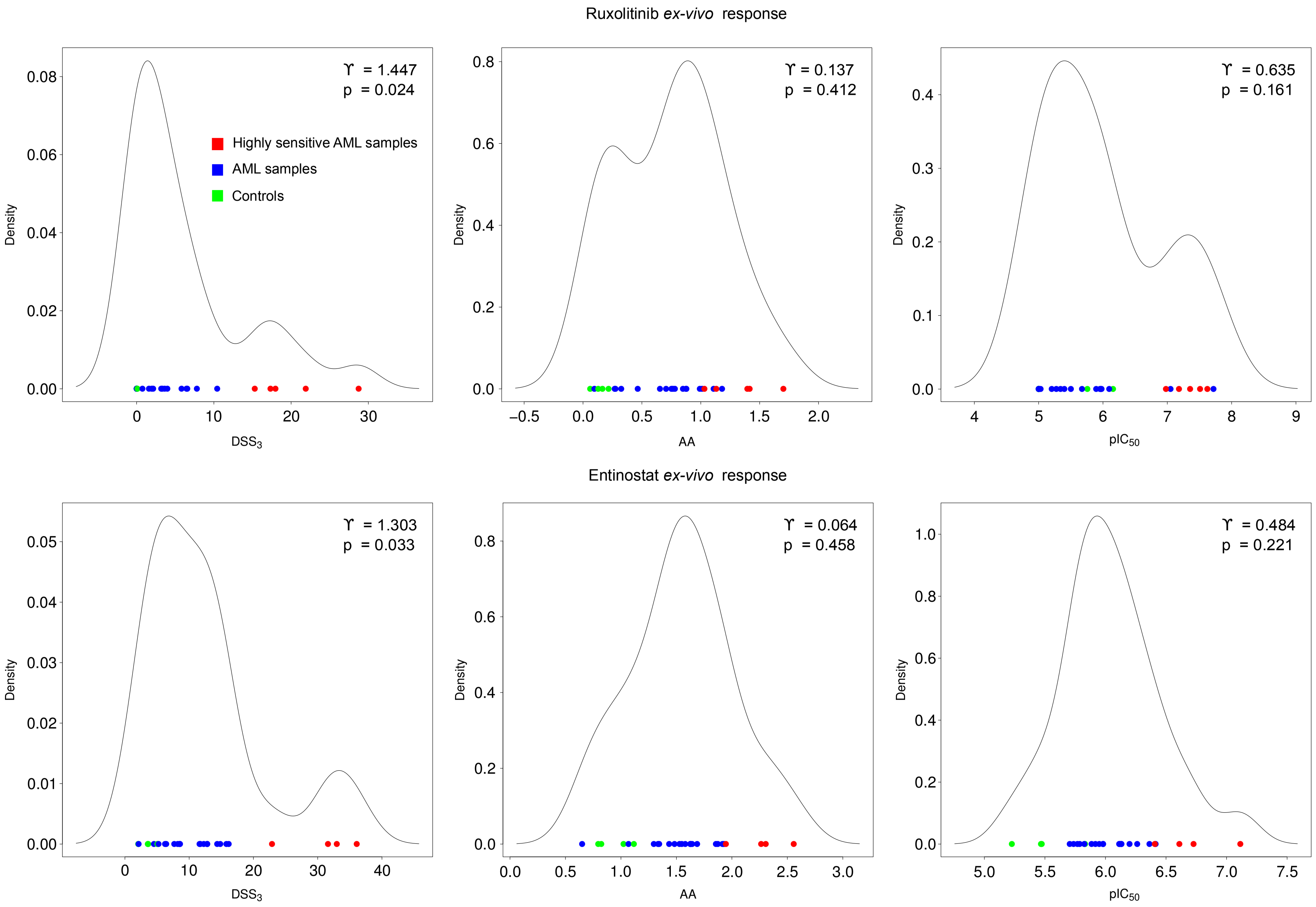

Supplementary Figure 4. Distribution of the response scores in the selected compound examples. Top: responses of the AML patient and control samples to ruxolitinib; Bottom, response of the AML patient and control samples to entinostat. Green points indicate the control samples and the red points the patient samples most sensitized to the compound. The observed skweness values ϒ and their significance levels are shown together with each distribution. Waterfall plots showing the individual responses to the two compounds are provided in Figure 5, and representative examples of the drug-response curves behind responsive and non-responsive cases are shown in Supplementary Figure 7.

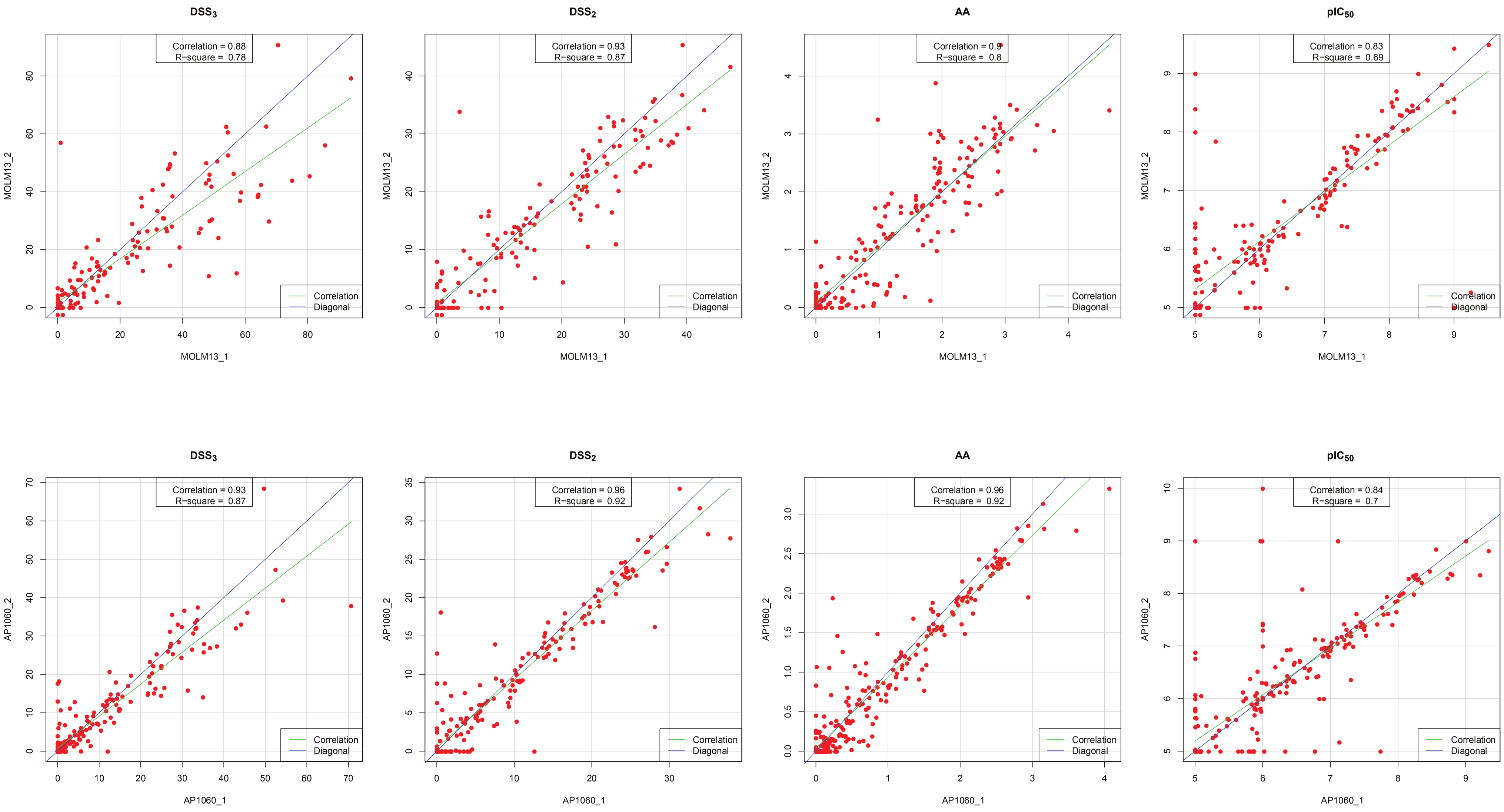

Supplementary Figure 5. Reproducibility of the response scores in terms of their profile correlation coefficient and coefficient of determination between technical replicates in two AML cell lines (MOLM13 and AP1060).

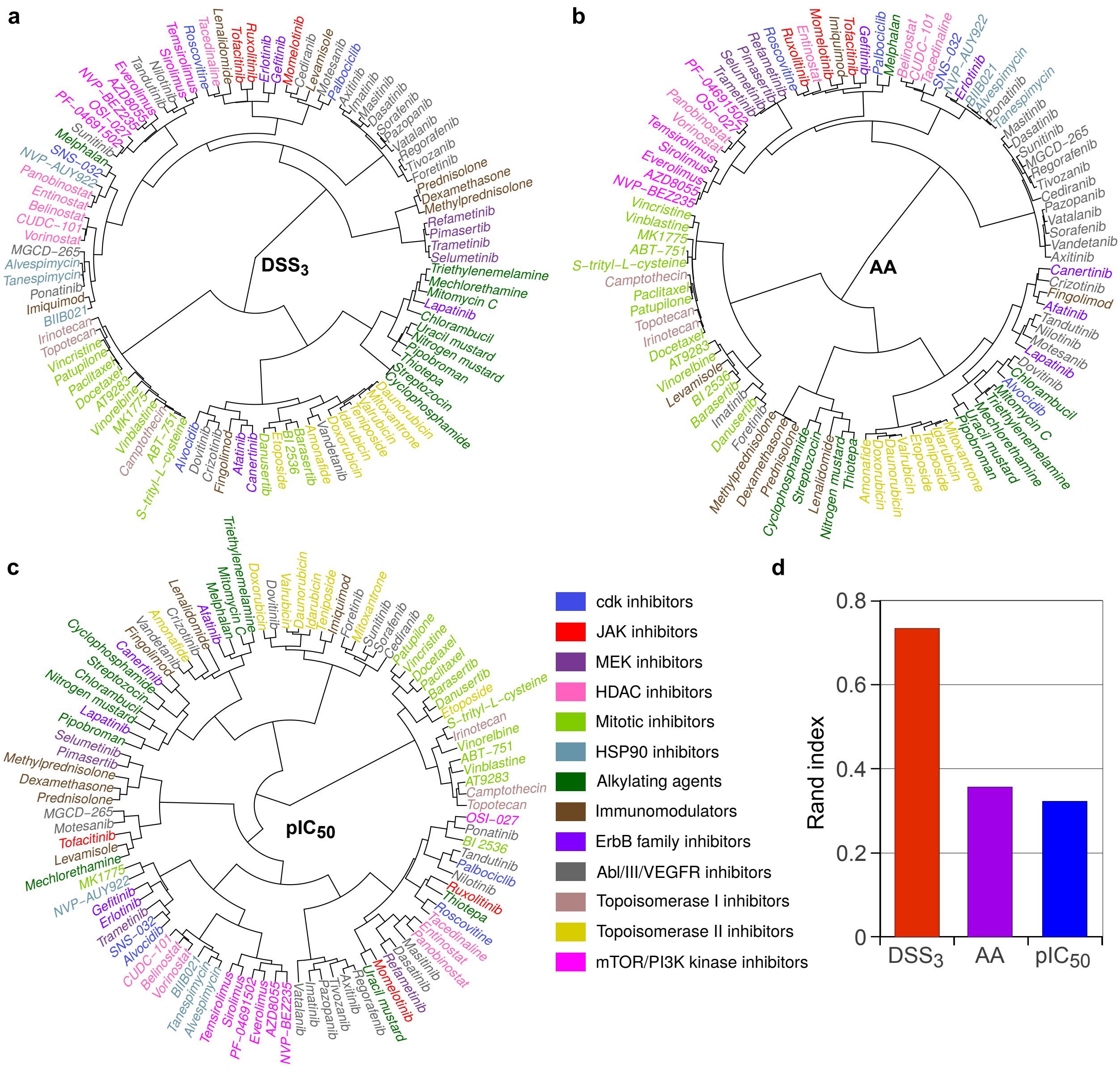

Supplementary Figure 6. Unsupervised clustering of the compounds based on their drug response profiles using the Ward's hierarchical clustering algorithm and Spearman's rank-based correlation coefficient. The primary mechanism of action (MoA) classification of the compounds is illustrated in color coding (Supplementary Table 1) (A) Clustering dendrogram of the DSS_3 drug response profiles over all the AML patient samples, relative to the control samples. (B) Clustering dendrogram of the AA drug response profiles over all the AML patient samples, relative to the control samples. (C) Clustering dendrogram of the pIC_{50} drug response profiles over all the AML patient samples, relative to the control samples. (D) Comparison of the response parameters in terms how accurately their compound clustering reflects the established MoA classes as assessed using adjusted Rand index.

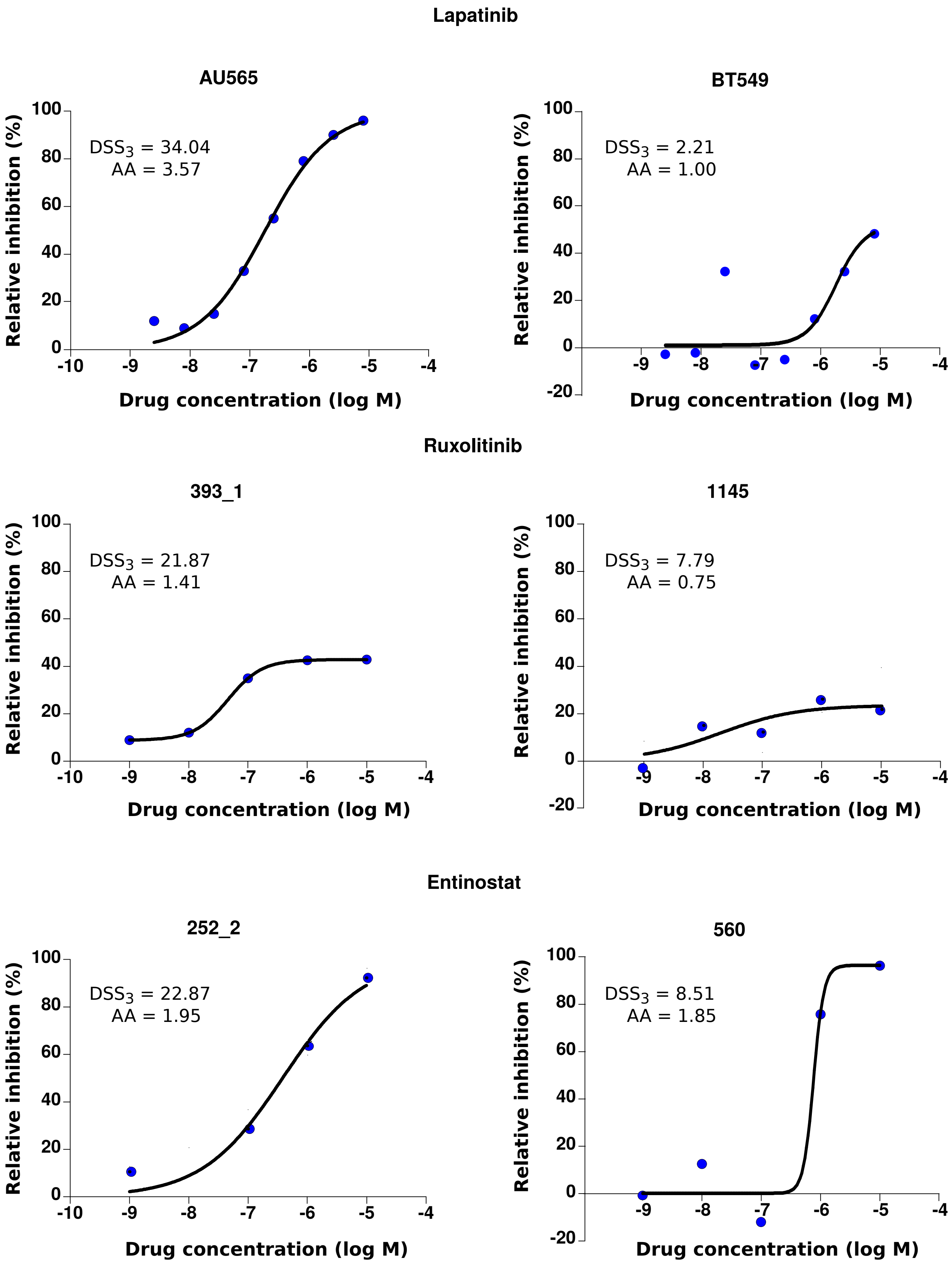

Supplementary Figure 7. Example drug-response curves behind the drug response patterns shownin waterfall plots Figures 4 and 5. DSS_3 version was implemented to favor clinically relevant response patterns that show potency over a wider therapeutic window (left), compared with marginally potent or toxic patterns that show activity at the highest dose levels only (right).

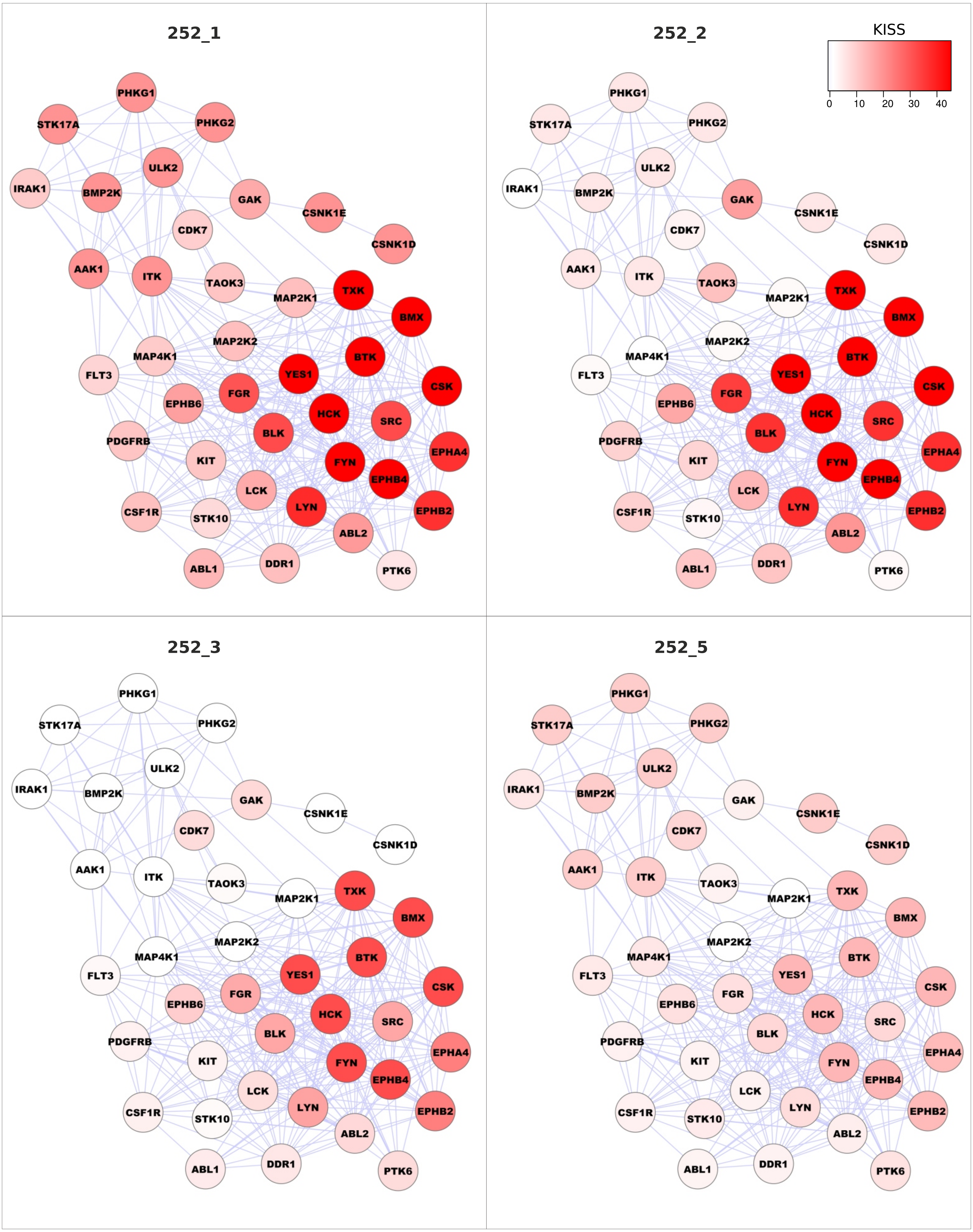

Supplementary Figure 8. Kinase addiction sub-network - "kinaddictome" – connecting those kinases the leukemia cells of an individual AML patient sample were most addicted to. Coloring indicates the degree of addiction to the selected kinase target sub-network (with kinase inhibition sensitivity score KISS > 5 in the sample 252_1), and edges connect kinase nodes with similar inhibitor specificity profiles in a recent large-scale biochemical screen (Spearman's rank-based correlation > 0.5; Davis et al. 2011). Separate panels show kinase networks constructed on the basis of the sample 1 (reflecting an initial therapeutic response), as well as for the later disease progression samples 2-5 (gradual development of resistance).

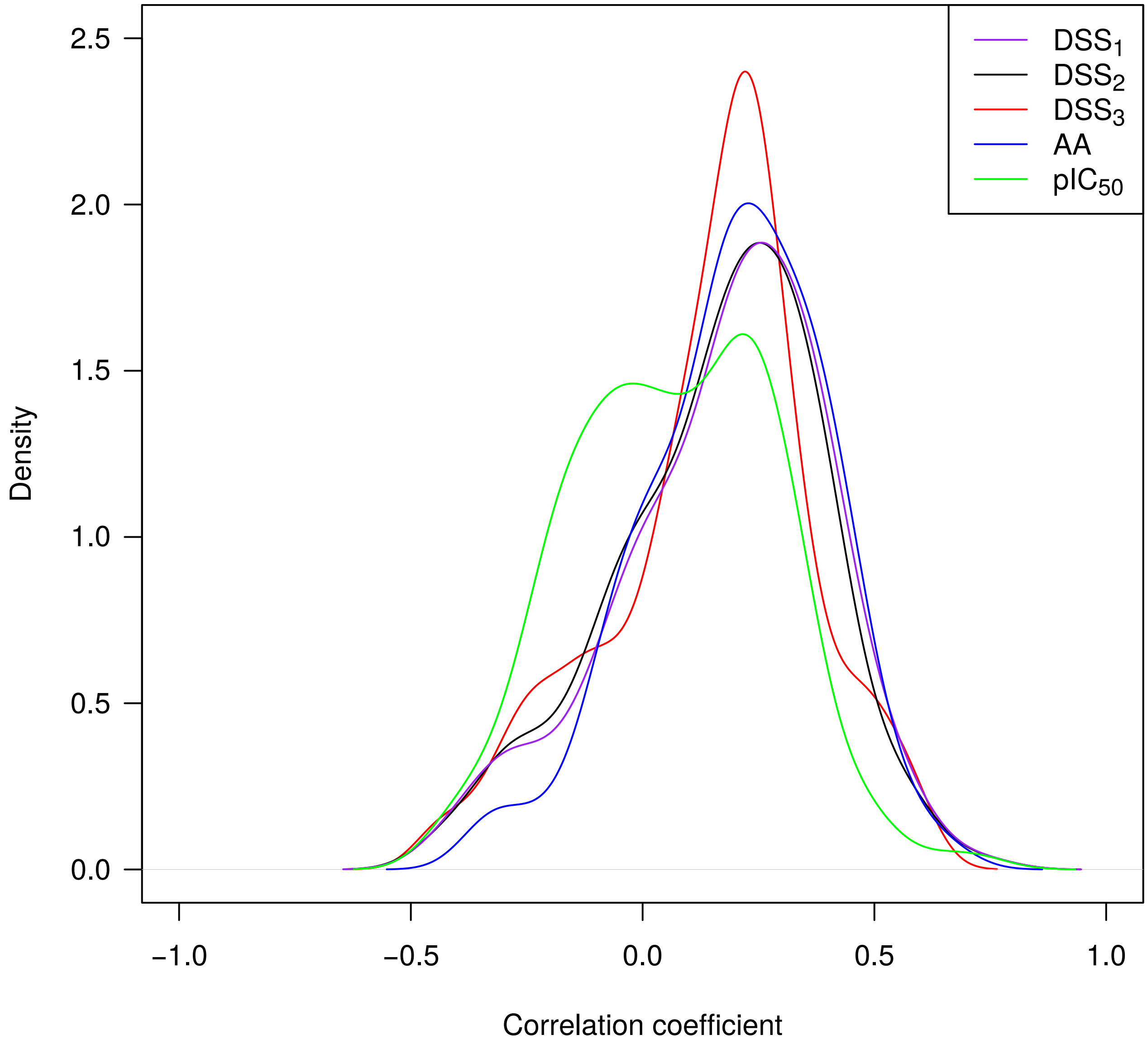

Supplementary Figure 9. Density distributions of correlation coefficients between blast counts and response metrics (DSS, AA, pIC_{50}). The distributions summarize correlations calculated over all the AML samples between their blast perctanges and response profiles.

Supplementary Table 1. FIMM panel of cancer compounds and their mechanism of action classification.

Compound	Aliases	Mechanism or cellular targets
Vincristine		Mitotic inhibitor
Vinblastine		Mitotic inhibitor
Paclitaxel		Mitotic inhibitor
BI 2536		Mitotic inhibitor
AT9283		Mitotic inhibitor
Danusertib		Mitotic inhibitor
ABT-751		Mitotic inhibitor
MK1775		Mitotic inhibitor
Barasertib	AZD1152-HQPA	Mitotic inhibitor
Patupilone	Epothilone B, EPO-906, EpoB	Mitotic inhibitor
Docetaxel	Taxotere, RP 56976	Mitotic inhibitor
Vinorelbine		Mitotic inhibitor
S-trityl-L-cysteine		Mitotic inhibitor
Palbociclib	PD-0332991	cdk inhibitor
SNS-032	BMS-387032	cdk inhibitor
Alvocidib	Flavopiridol, HMR-1275	cdk inhibitor
Roscovitine	Seliciclib, CYC202	cdk inhibitor
Imatinib	STI571	BCR/Abl/III/VEGFR inhibitor
Vatalanib	CGP-79787, PTK 787, ZK222584	BCR/Abl/III/VEGFR inhibitor
Dovitinib		BCR/Abl/III/VEGFR inhibitor
Crizotinib	PF-02341066, PF-2341066	BCR/Abl/III/VEGFR inhibitor
Foretinib	GSK1363089, XL880 ,EXEL-2880	BCR/Abl/III/VEGFR inhibitor
MGCD-265		BCR/Abl/III/VEGFR inhibitor
Ponatinib	AP24534	BCR/Abl/III/VEGFR inhibitor
Motesanib	AMG-706	BCR/Abl/III/VEGFR inhibitor
Cediranib		BCR/Abl/III/VEGFR inhibitor
Tivozanib	AV-951, KRN951	BCR/Abl/III/VEGFR inhibitor
Nilotinib	AMN-107	BCR/Abl/III/VEGFR inhibitor
Vandetanib	ZD6474	BCR/Abl/III/VEGFR inhibitor
Dasatinib	BMS-354825	BCR/Abl/III/VEGFR inhibitor
Axitinib	AG013736	BCR/Abl/III/VEGFR inhibitor
Pazopanib	GW786034, Armala	BCR/Abl/III/VEGFR inhibitor
Tandutinib		BCR/Abl/III/VEGFR inhibitor
Masitinib		BCR/Abl/III/VEGFR inhibitor
Sorafenib	Bay 43-9006, Nevaxar	BCR/Abl/III/VEGFR inhibitor
Sunitinib	Sutent, SU-11248	BCR/Abl/III/VEGFR inhibitor
Regorafenib	BAY73-4506	BCR/Abl/III/VEGFR inhibitor
Gefitinib		ErbB family inhibitor
Erlotinib		ErbB family inhibitor
Lapatinib		ErbB family inhibitor
Afatinib	BIBW2992	ErbB family inhibitor
Canertinib	CI-1033, PD 183805	ErbB family inhibitor
CUDC-101		HDAC inhibitor
Belinostat	PXD101, PX105684	HDAC inhibitor
Tacedinaline	Acetvldinaline. Gö 5549. PD 123654. C	HDAC inhibitor
Panobinostat	LBH-589, Faridak	HDAC inhibitor
Vorinostat	SAHA, Zolinza, MK-0683	HDAC inhibitor
Entinostat	SNDX-275, MS-275	HDAC inhibitor
Tanespimycin	17-AAG	HSP90 inhibitor
Alvespimycin	17-DMAG, KOS-1022	HSP90 inhibitor
BIIB021		HSP90 inhibitor
NVP-AUY922	AUY922	HSP90 inhibitor
Dexamethasone		Immunomodulatory
Imiquimod		Immunomodulatory
Levamisole		Immunomodulatory
Methylprednisolone		Immunomodulatory
Prednisolone		Immunomodulatory
Fingolimod		Immunomodulatory
Lenalidomide	CC-5013	Immunomodulatory
Selumetinib	AZD6244, ARRY-142886	MEK inhibitor
Pimasertib	MSC1936369B, AS703026	MEK inhibitor
Trametinib	GSK1120212	MEK inhibitor
Refametinib	BAY 869766, RDEA119	MEK inhibitor
Ruxolitinib	INCB018424	JAK inhibitor
Tofacitinib	CP-690550	JAK inhibitor
Momelotinib	LY2784544	JAK inhibitor
Camptothecin		topoisomerase I inhibitor
Topotecan		topoisomerase I inhibitor
Irinotecan	Camptosar	topoisomerase I inhibitor
Idarubicin		Topoisomerase II inhibitor
Mitoxantrone		Topoisomerase II inhibitor
Amonafide		Topoisomerase II inhibitor
Teniposide		Topoisomerase II inhibitor
Daunorubicin		Topoisomerase II inhibitor
Etoposide		Topoisomerase II inhibitor
Doxorubicin		Topoisomerase II inhibitor
Valrubicin		Topoisomerase II inhibitor
Chlorambucil		Alkylating agent
Cyclophosphamide		Alkylating agent
Melphalan		Alkylating agent
Mechlorethamine		Alkylating agent
Nitrogen mustard		Alkylating agent
Thiotepa		Alkylating agent
Pipobroman		Alkylating agent
Streptozocin		Alkylating agent
Triethylenemelamine		Alkylating agent
Mitomycin C		Alkylating agent
Uracil mustard		Alkylating agent
PF-04691502		mTOR/PI3K kinase inhibitor
AZD8055		mTOR/PI3K kinase inhibitor
OSI-027		mTOR/PI3K kinase inhibitor
Everolimus	RAD001, SDZ-RAD, Certican	mTOR/PI3K kinase inhibitor
Temsirolimus	Torisel, CCI-779	mTOR/PI3K kinase inhibitor
Sirolimus	Rapamycin	mTOR/PI3K kinase inhibitor
Dactolisib	BEZ235, NVP-BEZ235	mTOR/PI3K kinase inhibitor
Pilocarpine		
Zolendronic acid		
Anagrelide		
Tretinoin		
Goserelin		
Raloxifene		
Plicamycin		
Letrozole		
Bexarotene		
Celecoxib		
Anastrozole		
Lomustine		
Altretamine		
Amifostine		
Aminoglutethimide		
Thalidomide		
Dacarbazine		
Finasteride		
Fluorouracil		
Flutamide		
Ifosfamide		
Procarbazine		
Prednisone		
Mepacrine		
Temozolomide		
Fulvestrant		
Megestrol		
Tamoxifen		
Methotrexate		
Nilutamide		
Aminolevulinic acid		
Mitotane		
Allopurinol		
Busulfan		
Hydroxyurea		
Folinic acid		
Mercaptopurine		
Methoxsalen		
Thioguanine		
Carmustine		
Tacrolimus	FK-506	
Exemestane		
Navitoclax	ABT-263	
Obatoclax	GX15-070	
Abiraterone		
Serdemetan		
Decitabine	5-aza-2'-deoxycytidine	
Tipifarnib		
Tarenflurbil		
PF-477736		
AZD 7762		
Bimatoprost		
Doramapimod		
Bryostatin 1		
BMS-754807		
Idelalisib	CAL-101	
Erismodegib	LDE225,NVP-LDE225	
Fasudil		
Indibulin		
MK-2206		
Alisertib	MLN8237	
Nelarabine		
2-methoxyestradiol		
Plerixafor		
Vemurafenib		
XL-147		
YM155		
Linsitinib	OSI-906	
EMD1214063		
Lestaurtinib		
Saracatinib		
Bosutinib		
Enzastaurin	LY317615	
Pictilisib	GDC-0941	
Vismodegib	GDC-0449, HhAntag691	
Pentostatin		
Estramustine		
Floxuridine		
Gemcitabine		
Dactinomycin		
Cladribine		
Carboplatin		
Cisplatin		
Pemetrexed		
Oxaliplatin		
Cytarabine		
Dexrazoxone		
Arsenic trioxide		
Ixabepilone		
Azacitidine		
Clofarabine		
Fludarabine		
Bortezomib	PS-341, MS-341	
Bleomycin		
Quizartinib		
Prima-1 Met		
Carfilzomib		
AZ 3146		
Sotrastaurin		
XL765	SAR245409	
Midostaurin		
XAV-939		
UCN-01		
Ruboxistaurin	LY 333531	
Capecitabine		
Veliparib	ABT-888	
Rucaparib	AG-014699, AG-014447 ,PF-01367338	
Iniparib	BSI-201, IND-71677	
Olaparib	AZD2281, KU-0059436	

Supplementary Table 2. Predictive power of the scores to distinguish increasing activity classes form the inactive cases.

Comparison against inactives		Area under ROC curve (AUROC)				
**Activity class**	**Curves**	**AA**	**IC50**	**DSS1**	**DSS2**	**DSS3**
Inactive	612					
Low active	70	0.865	0.903	0.947	0.954	0.988
Semi active	65	0.962	0.953	0.988	0.990	0.998
Active	30	0.983	0.972	0.995	0.996	1.000
Very active	18	0.977	0.980	1.000	1.000	1.000
All active	183	0.930	0.940	0.971	0.978	0.995

Normalization of the DSS by the active dose range (DSS3) further increased power compared to the other scores ($p < 1E-07$)

Supplementary Table 3. Subset of CCLE hematopoietic and lymphoid cell lines, their RAS mutation status and sensitivity to PD−0325901 treatment.

CCLE cell line	Histology	Histology subtype	Gender	RAS mutation	Hot spot	DSS3	AA	pIC50
KO52	haematopoietic_neoplasm	acute_myeloid_leukaemia	Male	NRAS	p.G13R	66.146	5.074	8.602
HUT78	lymphoid_neoplasm	mycosis_fungoides-Sezary_syndrome	Male	NRAS	p.Q61K	62.720	5.161	7.833
HDMYZ	lymphoid_neoplasm	Hodgkin_lymphoma		NRAS	p.G13D	62.632	4.690	7.756
KARPAS620	lymphoid_neoplasm	plasma_cell_myeloma	Female	KRAS	p.G12D	59.734	4.076	8.327
P31FUJ	haematopoietic_neoplasm	acute_myeloid_leukaemia	Male	NRAS	p.G12C	56.903	4.567	7.948
AMO1	lymphoid_neoplasm	plasma_cell_myeloma	Female	KRAS	p.A146T	34.212	2.992	7.344
KHM1B	lymphoid_neoplasm	plasma_cell_myeloma	Male	KRAS	p.G12C	32.007	3.081	8.250
SUDHL10	lymphoid_neoplasm	diffuse_large_B_cell_lymphoma	Male	KRAS	p.I171M	26.680	1.843	8.072
P12ICHIKAWA	lymphoid_neoplasm	acute_lymphoblastic_T_cell_leukaemia	Male	NRAS	p.G12D	24.468	2.486	7.568
KASUMI2	lymphoid_neoplasm	acute_lymphoblastic_B_cell_leukaemia	Male	KRAS	p.V14L	22.706	2.199	8.020
MOLP8	lymphoid_neoplasm	plasma_cell_myeloma	Male	KRAS/NRAS	p.Q61L	21.416	2.959	7.326
KMM1	lymphoid_neoplasm	plasma_cell_myeloma	Male	KRAS/NRAS	p.V9I, p.G13D	20.099	2.354	6.886
697	lymphoid_neoplasm	acute_lymphoblastic_B_cell_leukaemia	Male	NRAS	p.G12D	16.828	2.586	7.492
MINO	lymphoid_neoplasm	mantle_cell_lymphoma	Male	NRAS	p.G13D	8.726	0.249	7.335
ALLSIL	lymphoid_neoplasm	acute_lymphoblastic_T_cell_leukaemia	Male	KRAS	intron	8.417	1.730	6.974
TOLEDO	lymphoid_neoplasm	diffuse_large_B_cell_lymphoma	Female	KRAS	p.G13D	1.470	0.283	5.173
L363	lymphoid_neoplasm	plasma_cell_myeloma	Female	NRAS	p.Q61H	0.000	1.458	5.097
RPMI8402	lymphoid_neoplasm	acute_lymphoblastic_T_cell_leukaemia	Male	HRAS	p.A134S	0.000	0.466	5.097
OCIAML2	haematopoietic_neoplasm	acute_myeloid_leukaemia		WT		94.014	7.280	8.602
SIGM5	haematopoietic_neoplasm	acute_myeloid_leukaemia		WT		92.693	7.381	8.602
KU812	haematopoietic_neoplasm	chronic_myeloid_leukaemia		WT		59.094	4.571	7.520
EM2	haematopoietic_neoplasm	blast_phase_chronic_myeloid_leukaemia		WT		55.926	4.539	7.725
F36P	haematopoietic_neoplasm	acute_myeloid_leukaemia		WT		48.405	3.785	8.482
BDCM	lymphoid_neoplasm	acute_lymphoblastic_B_cell_leukaemia		WT		40.419	2.320	7.073
KCL22	haematopoietic_neoplasm	blast_phase_chronic_myeloid_leukaemia		WT		35.874	3.692	7.507
NCO2	haematopoietic_neoplasm	blast_phase_chronic_myeloid_leukaemia		WT		35.713	3.948	6.820
OCIAML5	haematopoietic_neoplasm	acute_myeloid_leukaemia		WT		31.851	3.352	6.991
JVM3	lymphoid_neoplasm	chronic_lymphocytic_leukaemia-small_lymphocytic_ly		WT		30.114	3.464	7.877
MEG01	haematopoietic_neoplasm	blast_phase_chronic_myeloid_leukaemia		WT		27.666	3.332	6.580
SKMM2	lymphoid_neoplasm	plasma_cell_myeloma		WT		24.696	2.113	7.365
KG1	haematopoietic_neoplasm	acute_myeloid_leukaemia		WT		23.272	3.257	6.595
MONOMAC1	haematopoietic_neoplasm	acute_myeloid_leukaemia		WT		22.301	3.630	7.630
MEC1	lymphoid_neoplasm	chronic_lymphocytic_leukaemia-small_lymphocytic_ly		WT		17.251	2.216	8.130
KARPAS299	lymphoid_neoplasm	anaplastic_large_cell_lymphoma		WT		13.621	1.469	6.707
OPM2	lymphoid_neoplasm	plasma_cell_myeloma		WT		12.450	1.396	7.726
SUPM2	lymphoid_neoplasm	anaplastic_large_cell_lymphoma		WT		10.700	1.519	6.422
DEL	lymphoid_neoplasm	NS		WT		8.451	1.312	7.393
U937	lymphoid_neoplasm	diffuse_large_B_cell_lymphoma		WT		8.076	1.514	6.116
KMS26	lymphoid_neoplasm	plasma_cell_myeloma		WT		6.675	1.505	5.135
PFEIFFER	lymphoid_neoplasm	diffuse_large_B_cell_lymphoma		WT		5.551	0.890	7.097
KE97	lymphoid_neoplasm	plasma_cell_myeloma		WT		4.603	0.447	7.002
GRANTA519	lymphoid_neoplasm	mantle_cell_lymphoma		WT		4.601	1.272	6.795
EB1	lymphoid_neoplasm	Burkitt_lymphoma		WT		4.258	0.920	7.126
CMK86	haematopoietic_neoplasm	acute_myeloid_leukaemia		WT		3.592	1.799	7.639
OCILY10	lymphoid_neoplasm	diffuse_large_B_cell_lymphoma		WT		1.143	0.276	5.202
P3HR1	lymphoid_neoplasm	Burkitt_lymphoma		WT		0.807	0.298	5.471
HEL9217	haematopoietic_neoplasm	acute_myeloid_leukaemia		WT		0.525	0.592	6.198
MC116	lymphoid_neoplasm	B_cell_lymphoma_unspecified		WT		0.229	0.166	5.578
KARPAS422	lymphoid_neoplasm	diffuse_large_B_cell_lymphoma		WT		0.000	0.813	8.097
MOLT16	haematopoietic_neoplasm	acute_myeloid_leukaemia		WT		0.000	1.740	7.598
HT	lymphoid_neoplasm	B_cell_lymphoma_unspecified		WT		0.000	0.924	7.268
KMS12BM	lymphoid_neoplasm	plasma_cell_myeloma		WT		0.000	1.344	5.883
LP1	lymphoid_neoplasm	plasma_cell_myeloma		WT		0.000	1.737	5.097
SUDHL8	lymphoid_neoplasm	diffuse_large_B_cell_lymphoma		WT		0.000	1.511	5.097
KMS11	lymphoid_neoplasm	plasma_cell_myeloma		WT		0.000	1.191	5.097
DOHH2	lymphoid_neoplasm	diffuse_large_B_cell_lymphoma		WT		0.000	0.878	5.097
SUDHL6	lymphoid_neoplasm	diffuse_large_B_cell_lymphoma		WT		0.000	0.853	5.097
JURKAT	lymphoid_neoplasm	acute_lymphoblastic_T_cell_leukaemia		WT		0.000	0.717	5.097
HH	lymphoid_neoplasm	adult_T_cell_lymphoma-leukaemia		WT		0.000	0.711	5.097
SUDHL4	lymphoid_neoplasm	diffuse_large_B_cell_lymphoma		WT		0.000	0.698	5.097
CI1	lymphoid_neoplasm	B_cell_lymphoma_unspecified		WT		0.000	0.604	5.097
RAJI	lymphoid_neoplasm	Burkitt_lymphoma		WT		0.000	0.531	5.097
SUPT1	lymphoid_neoplasm	acute_lymphoblastic_T_cell_leukaemia		WT		0.000	0.464	5.097
KMS34	lymphoid_neoplasm	plasma_cell_myeloma		WT		0.000	0.329	5.097
JM1	lymphoid_neoplasm	B_cell_lymphoma_unspecified		WT		0.000	0.291	5.097
BL70	lymphoid_neoplasm	Burkitt_lymphoma		WT		0.000	0.067	5.097
EB2	lymphoid_neoplasm	Burkitt_lymphoma		WT		0.000	0.048	5.097
L428	lymphoid_neoplasm	Hodgkin_lymphoma		WT		0.000	0.012	5.097
BL41	lymphoid_neoplasm	Burkitt_lymphoma		WT		0.000	0.000	5.097
MJ	lymphoid_neoplasm	mycosis_fungoides-Sezary_syndrome		WT		0.000	0.000	5.097
REH	lymphoid_neoplasm	acute_lymphoblastic_B_cell_leukaemia		WT		0.000	0.000	5.097

The cell lines highlighted in green were most sensitive to the PD−0325901 treatment both in RAS mutated and WT cells

Supplementary Table 4. Subset of CCLE breast cancer cell lines, their ERBB2 status and sensitivity to lapatinib and erlotinib treatments.

CCLE	ERBB2 status	ERBB2	Labatinib response			Erlotinib response		
Cell line	Subtype	Expression	AA	pIC50	DSS3	AA	pIC50	DSS3
BT474_BREAST	Luminal, ERBB2 AMP	11.753	3.381	6.975	42.139	0.316	5.706	0.860
AU565_BREAST	Luminal, ERBB2 AMP	12.997	3.568	6.742	34.050	0.491	5.485	0.619
ZR7530_BREAST	Luminal, ERBB2 AMP	12.944	3.090	6.880	33.727	0.040	8.636	0.000
SKBR3_BREAST	Luminal, ERBB2 AMP	11.993	2.905	6.901	27.413	0.648	5.724	1.049
MDAMB175VII_BREAST	Luminal	9.674	2.360	6.337	17.934	1.196	5.773	4.334
MDAMB453_BREAST	Luminal	10.005	1.582	5.858	13.414	0.147	5.001	0.112
HCC1954_BREAST	Basal	12.629	2.076	5.941	12.142	0.840	5.811	2.734
CAL851_BREAST	Basal	7.827	1.905	6.158	10.968	2.023	6.239	15.595
UACC812_BREAST	Luminal, ERBB2 AMP	11.659	1.318	6.570	10.296	0.443	5.522	0.712
HCC1806_BREAST	Basal	7.352	1.437	6.375	8.471	2.100	5.596	7.660
HDQP1_BREAST	Basal	7.479	2.469	6.283	6.671	1.637	6.795	10.503
MDAMB468_BREAST	Basal	6.451	1.417	5.658	3.954	1.865	5.798	9.253
MCF7_BREAST	Luminal	7.916	0.424	5.488	3.446	0.357	5.672	0.824
MB157_BREAST	Basal	6.556	1.283	5.935	2.793	1.436	6.275	3.807
BT549_BREAST	Basal	6.603	1.000	5.745	2.207	0.247	5.000	0.038
T47D_BREAST	Luminal	8.231	0.573	6.148	2.011	0.179	5.000	0.042
HCC1395_BREAST	Basal	6.233	0.347	6.028	1.620	0.552	5.558	0.568
BT20_BREAST	Basal	8.032	0.668	5.527	0.844	1.308	5.000	3.498
MDAMB415_BREAST	Luminal	7.414	0.495	5.761	0.736	0.035	5.568	0.028
EFM19_BREAST	Luminal	8.968	0.755	5.048	0.433	0.099	5.000	0.000
HCC70_BREAST	Basal	7.499	0.607	5.065	0.318	0.341	6.680	3.033
CAMA1_BREAST	Luminal	7.851	0.146	5.574	0.297	0.004	5.785	0.000
HMC18_BREAST	Basal	6.364	0.370	5.372	0.220	0.348	5.995	0.000
HS578T_BREAST	Basal	6.990	0.203	5.602	0.210	0.058	5.470	0.025
HCC1569_BREAST	Basal, ERBB2 AMP	12.101	0.318	5.000	0.198	0.189	5.000	0.002
MDAMB436_BREAST	Basal	6.255	0.246	6.224	0.133	0.428	5.000	0.306
		Expression correlation	0.66059	0.45880	0.74006			
		P-value	**0.00024**	**0.01840**	**0.00002**			

The cell lines highlighted in yellow were selected based on the multimodal DSS3 distribution, where these four lines were highly sensitive to labatinib
